# Gα_12_ overexpression in hepatocytes by ER stress exacerbates acute liver injury via ROCK1-mediated miR-15a and ALOX12 dysregulation

**DOI:** 10.7150/thno.67722

**Published:** 2022-01-09

**Authors:** Jihoon Tak, Yun Seok Kim, Tae Hyun Kim, Gil-Chun Park, Shin Hwang, Sang Geon Kim

**Affiliations:** 1 College of Pharmacy, Seoul National University, Seoul 08826, Republic of Korea; 2 Department of Clinical Pharmacology and Therapeutics, Seoul National University College of Medicine, Seoul 03080, Republic of Korea; 3 Joslin Diabetes Center, Harvard Medical School, Boston, MA 02215, USA; 4 Drug Information Research Institute, College of Pharmacy, Sookmyung Women's University, Seoul 04310, Republic of Korea; 5 Department of Surgery, Asan Medical Center, University of Ulsan, College of Medicine, Seoul, Republic of Korea; 6 College of Pharmacy and Integrated Research Institute for Drug Development, Dongguk University-Seoul, Goyang-si, Kyeonggi-do 10326, Republic of Korea

**Keywords:** Gα_12_, acetaminophen-induced liver injury, miR-15a, ALOX12, GPX4

## Abstract

**Rationale:** Liver injury must be further characterized to identify novel therapeutic approaches. Endoplasmic reticulum (ER) stress may cause hepatocyte death. Gα_12_ affects cell viability and its expression varies depending on physiological conditions. This study investigated whether hepatocyte-specific Gα_12_ overexpression affects acute liver injury, and if so, what the underlying mechanisms and treatment strategies are.

**Methods:** All experiments were performed using human liver, hepatocytes, and toxicant injury models with *Gna12* KO and/or hepatocyte-specific Gα_12_ overexpression. RNA-sequencing, immunoblotting, immunohistochemistry, reporter assays, and mutation assays were conducted.

**Results:** Hepatic Gα_12_ was overexpressed in mice challenged with acetaminophen or other ER stress inducers or in patients with acute liver injury or fibrosis/cirrhosis. Several Gα_12_ and ER-associated pathways were identified using transcriptomic analysis. Acetaminophen intoxication was characterized by lipid peroxide-induced ferroptosis and was less severe in Gα_12_-deficient animals and cells. Conversely, Gα_12_ overexpression in wild-type or *Gna12* KO hepatocytes increased hepatotoxicity, promoting lipid peroxidation, inflammation, and ferroptosis. IRE1α-dependent Xbp1 transactivated *Gna12*. Moreover, Gα_12_ overexpression enhanced the ability of acetaminophen to induce ALOX12, while downregulating GPX4. The level of miR-15a, herein identified as an ALOX12 inhibitor, was decreased. siRNA knockdown or pharmacological inhibition of ROCK1 prevented dysregulation of ALOX12 and GPX4, rescuing animals from toxicant-induced ferroptosis. These changes or correlations among the targets were confirmed in human liver specimens and datasets of livers exposed to other injurious medications.

**Conclusions:** Gα_12_ overexpression by ER stress facilitates hepatocyte ferroptosis through ROCK1-mediated dysregulation of ALOX12, and miR-15a, supporting the concept that inhibition of Gα_12_ overexpression and/or ROCK1 axis may constitute a promising strategy for acute liver injury.

## Introduction

Despite individual variabilities, the mortality rate of patients admitted to hospitals due to drug-induced liver injury is significant (~10%). Hepatotoxicants, herbal medicines, and the metabolites from some medications predispose patients to severe liver toxicity, particularly when they present preexisting conditions such as alcoholic liver disease, cirrhosis, cholestasis, or energy deficiency. Moreover, acetaminophen (APAP) hepatotoxicity, which accounts for ~50% of acute liver failure occurrences in Western societies, is a major cause of acute liver injury, and other toxicants also cause extensive liver damage, which may lead to uncontrolled liver injury 1, 2. Given the public concern regarding drug/toxicant intoxications, many efforts have been made to understand the basis of acute liver injury, as well as to identify potential treatment agents.

Gα_12_ converges signals from G-protein-coupled receptors and its expression varies depending on physiological conditions. Moreover, Gα_12_ may play cell type-specific roles in different pathologies by amplifying or inhibiting receptor signals depending on their expression level. Gα_12_ regulates SIRT1-dependent mitochondrial respiration, and thus its overexpression contributes to mitochondrial fuel oxidation. In our other studies, phosphorylated JNK levels in macrophages and palmitate-induced apoptosis were diminished in deficient of Gα_12_ 3, 4. Due to the divergent roles of Gα_12_ in energy metabolism and different cell type-specific behaviors, we sought to investigate whether the Gα_12_ signaling axis exerts a deleterious or protective effect on acute liver injury using mouse models.

Endoplasmic reticulum (ER) stress occurs due to an accumulation of unfolded or misfolded proteins in the ER. A cascade of events may be activated if the aforementioned ER stress exceeds the ER folding capacity, thus resulting in irreversible damage or cell death 5, 6. Therefore, developing effective strategies to manage ER stress is a key step to control hepatitis development or the progression of different fibrosis stages 7, 8. Despite the contribution of ER stress in the development of injurious liver diseases, the molecular mechanisms of ER stress regulation in hepatocytes, particularly during the progression of acute liver injury, have not been fully elucidated.

Ferroptosis is characterized by excessive production of lipid peroxides mediated by iron catalysis and is distinct from apoptosis, necroptosis, and pyroptosis. Also, it occurs when intracellular glutathione peroxidase 4 (GPX4) is inhibited by a lowered GSH level 9. Inhibition of GPX4 causes the accumulation of lipid peroxides derived from polyunsaturated fatty acid-containing phospholipids, triggering cell membrane damage and eventually cell death in the absence of specific effector molecules 10. Lipid peroxides are normally removed by cellular sulfhydryls, which convert them to lipid alcohols. Thus, depletion of glutathione promotes toxicant-induced liver injury. Moreover, ferroptosis has been implicated in other pathological processes including neurodegenerative disease, cancer, and kidney diseases 11, 12. Nonetheless, the regulators responsible for ferroptosis in hepatocytes should be further characterized in the context of identifying novel treatment approaches.

Given the importance of ferroptosis in cell fate determination and the role of hepatocytes as a major cell type for iron storage, this study focused on the effect of the Gα_12_ pathway on acute liver injury in the context of ferroptosis. Here, we report that ER stress triggers Gα_12_ overexpression, which then facilitates the production of lipid peroxides for ferroptosis through the induction of ALOX12, as mediated by miR-15a dysregulation. Further, we sought to identify new strategies for the treatment of hepatic intoxication. Specifically, we propose that pharmacological inhibition or siRNA-induced knockdown of ROCK1 may effectively prevent ferroptosis downstream from Gα_12_. To confirm the effects of liver-specific Gα_12_ overexpression, we utilized a lentiviral albumin-Gα_12_ (Lv-Alb-Gα_12_) delivery system in WT or *Gna12* KO animals subjected to APAP challenge and found more severe hepatic injuries and inflammatory responses. Finally, the alterations in the levels of the targets discovered in this study were corroborated using liver specimens from patients with acute liver injury or fibrosis/cirrhosis, thus highlighting the potential applications of the Gα_12_-ROCK1 axis for the treatment of liver injury, and the resulting fibrosis and cirrhosis in humans.

## Materials and methods

Additional details of the materials and experimental protocols are available in the Supplementary Materials and Methods.

### Animal models and experiments

The mice were housed in a 12 h light/dark cycle and relative humidity of 50% ± 5% under filtered, pathogen-free air, with food and water available *ad libitum*. Details of the generation of the *Gna12* KO mice used in this study have been described previously 13. Male mice at 8 to 12 weeks of age, unless otherwise indicated, were used. To minimize environmental differences, mice were maintained for at least a week before each experiment. For the acute liver injury model, male C57BL/6 mice were fasted overnight prior to a single dose of APAP treatment (300 mg/kg BW, i.p.), and the liver and blood samples were obtained 6 h afterward; In this experiment, we chose a 6 h time point because APAP treatment at the above dose results in increases of serum ALT activities up to 24 h, which peaked at 6 h post-treatment 14. The sampling protocol was applied in *Gna12* KO mice and their age-matched WT littermates. Where indicated, mice were given an injection of ferrostatin-1 (1 mg/kg BW, i.p.) 1 h before APAP treatment. Carbon tetrachloride (CCl_4_, 0.5 mL/kg BW, 1:20 in corn oil, i.p.) was injected twice a week for the indicated time period (2 or 6 weeks) for another liver injury model. Separately, male C57BL/6 mice were fasted overnight before a single dose of BSO treatment (1 g/kg BW, i.p.), and tissue and blood samples were obtained 6 h later. To induce ER stress, male C57BL/6 mice were injected with a single dose of tunicamycin (Tm; 2 mg/kg, i.p.) and were subjected to analyses 72 h afterward. For an *in vivo* rescue experiment, male C57BL/6 mice were fasted overnight and treated with a single dose of APAP (300 mg/kg BW, i.p.). After 1 h, the mice were exposed to ripasudil (50 mg/kg BW, i.p.) and tissue samples were obtained 5 h afterward.

### Statistical analyses

Statistical significance was tested via two-tailed Student's t-tests, Mann-Whitney U test, or one-way ANOVA coupled with Bonferroni's method, Tukey's honestly significant difference test, or the least significant difference multiple comparison procedure, where appropriate. Coefficients of correlation (r) were determined via the Pearson correlation method. Differences were considered significant at *P* < 0.05. Statistical analyses were performed using IBM SPSS Statistics 26 software or Prism version 8.0 (GraphPad Software).

### Study approval

All animal studies were approved by the institutional review board of the Seoul National University and conducted under the guidelines of the Institutional Animal Care and Use Committee (IACUC) at Seoul National University. Human liver samples were obtained from donors and recipients undergoing liver transplantation from 2011 to 2020 after histologic examination and ultrasonography at Asan Medical Center (Seoul, South Korea) (IRB no. 2021-0839). All patients in this study provided written informed consent, and the study was approved by the institutional review board of Asan Medical Center.

## Results

### Association between Gα_12_ overexpression and ER stress in response to APAP toxicity

**Figure 1:**
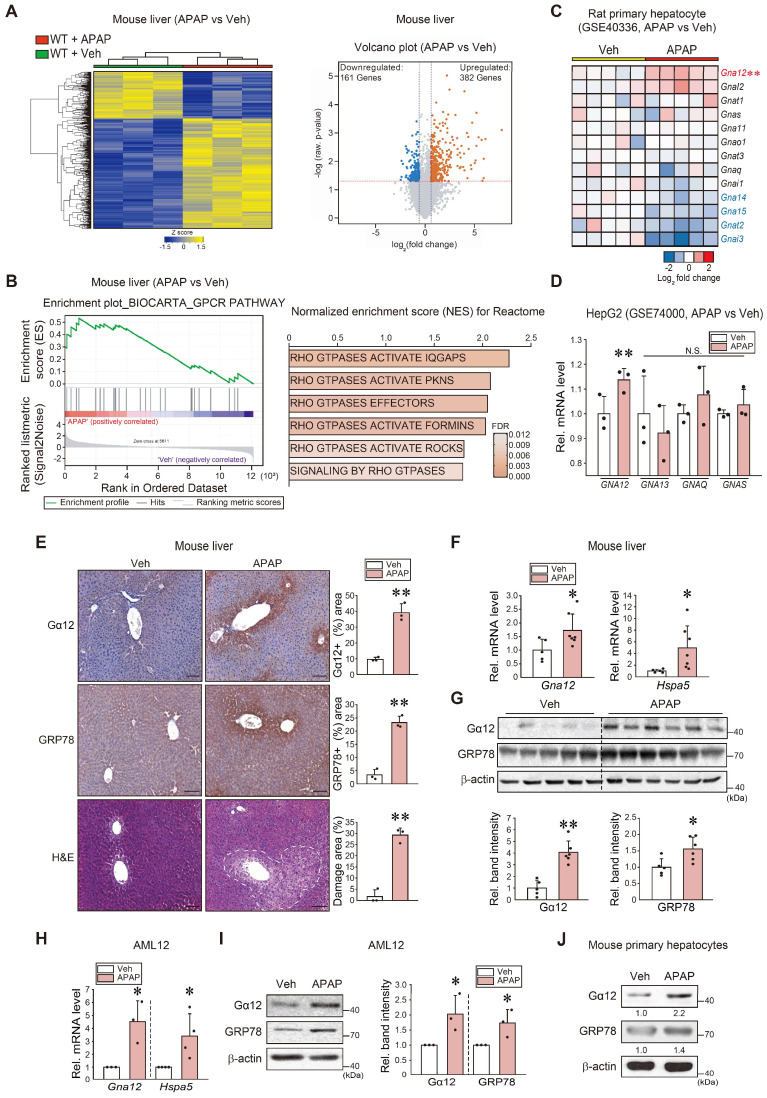
** APAP-induced Gα_12_ overexpression in hepatocytes in association with the unfolded protein response. (A)** Heatmap and hierarchical correlation analysis for differentially expressed genes in liver tissue between vehicle- and APAP-treated mice using RNA-seq data (left, n = 3 each). Volcano plot of RNA-seq data. Horizontal and vertical lines indicate the filtering criteria (absolute fold-change ≥ 1.5 and *P* < 0.05, respectively). Red and blue dots show differentially expressed genes (DEGs) upregulated (382 genes) or downregulated (161 genes) in response to APAP treatment (right, n = 3 each). **(B)** GSEA enrichment plot of the Biocarta category showing the GPCR pathway (left, NES = 1.6, FDR < 0.1) and Rho/ROCK-related signaling pathways positively correlated with APAP-exposed WT mice (300 mg/kg BW, 6 h) in the Reactome pathway (right, n = 3 each). NES and FDR are shown in the bar graph. **(C)** Heatmap of *Gna* subunits in primary hepatocytes treated with APAP or vehicle using microarray dataset (n = 5 each). **(D)** The levels of major *GNA* transcripts in HepG2 cells treated with APAP (2 mM, 24 h) relative to vehicle control derived from a public dataset (GSE74000). **(E)** Gα_12_ and GRP78 immunohistochemistry and histopathology analyses in mouse liver samples (H&E; scale bar: 200 μm) (left). Percent areas of Gα_12_, GRP78, and damaged areas were assessed using the Image J program (right). The mice were injected with a single dose of APAP (300 mg/kg BW, 6 h) (n = 3 each). **(F)** qRT-PCR assays for *Gna12* and *Hspa5* in APAP-treated WT mice (n = 5 or 7 each). **(G)** Immunoblottings (upper) for Gα_12_ and GRP78 in APAP-treated WT mice. The band intensities represent values relative to each respective control (lower) (n = 5 or 6 each). **(H)** qRT-PCR assays for *Gna12* and *Hspa5* in AML12 cells treated with APAP (10 mM, 12 h) (n = 3 or 4 each). **(I)** Immunoblottings (left) for Gα_12_ and GRP78 in AML12 cells treated with APAP (10 mM, 12 h). The band intensities represent values relative to each respective control (right, n = 3 or 4 each). **(J)** Immunoblottings for Gα_12_ and GRP78 in mouse primary hepatocytes treated with APAP (10 mM, 12 h) (n = 3 each). For D-I, the reported values represent mean ± SD (*P < 0.05, **P < 0.01). Statistical significance was tested via two-tailed Student's *t*-tests.

To examine the gene pathways affected by liver intoxication, we first performed RNA-sequencing (RNA-seq) analysis using liver tissues of mice treated with an excessive dose of APAP (300 mg/kg BW, i.p.) and found that APAP treatment induced a marked shift in the transcriptional profile (Figure 1A, left). Specifically, a total of 382 genes were significantly upregulated, whereas 161 genes were downregulated (Figure 1A, right). Moreover, a significant proportion of dysregulated genes were associated with the 'GPCR pathway', which was ranked within the top 25 most enriched gene sets in APAP-treated mice compared to controls (Figure 1B, left). Particularly, gene sets associated with Rho/ROCK pathways downstream from Gα_12_ showed significant enrichment in APAP-treated mice (Figure 1B, right). Next, we assessed the gene ontology (biological processes) of primary hepatocytes using the GSE40336 dataset; APAP treatment upregulated the genes associated with intracellular signal transduction and regulation of Rho protein signal transduction among five major pathways (Figure S1A). Given that the G protein level varies in different cell types and organs, we assessed major G-protein levels. Among the major Gα subunits examined herein, *Gna12* transcript levels were highly upregulated (Figure 1C). A similar result was obtained when analyzing a different dataset (GSE74000), indicating the ability of APAP to induce* GNA12* (Figure 1D).

Interestingly, gene ontology (GO) and biological process analyses of another dataset obtained from mice livers (GSE104302) confirmed that APAP treatment upregulated the expression of UPR-associated genes, as well as those linked to the “epigenetic process” and “chaperone-mediated protein folding cofactor” pathways (Figure S1B). In subsequent experiments, WT C57BL/6 mice exposed to a single dose of APAP exhibited a distinctly enhanced Gα_12_ staining intensity, particularly around the central vein area. A similar staining pattern was observed for GRP78. Histopathological analysis confirmed the occurrence of pronounced hepatocyte injuries (Figure 1E). Increases in *Gna12* and *Hspa5* transcript levels were confirmed (Figure 1F). Moreover, immunoblotting confirmed the ability of APAP to induce Gα_12_ (Figure 1G). Furthermore, increases in Gα_12_ and GRP78 expression were verified using hepatocyte models (Figure 1H-J). Lastly, we identified correlations between *Gna12* and ER stress marker (*Ddit3*) levels using datasets from human primary hepatocytes and mouse livers with liver injury (tolvaptan- and diclofenac-induced, respectively) (Figure S1C). Additionally, Gα_12_ expression was upregulated in the liver of mice repeatedly exposed to CCl_4_ (a known ER stress-inducing toxicant) (Figure S1D) 15.

To assess the effects of ER stress on Gα_12_, we analyzed another dataset from mice treated with tunicamycin (Tm) and found that ER stress increases and positively correlated with the level of *Gna12* transcripts (not *Gna13* or others) in the liver (Figure S1E). Taken together, these results support the hypothesis that liver intoxication results in Gα_12_ overexpression in hepatocytes via the ER stress response.

### IRE1α-mediated Xbp1s-dependent induction of Gα_12_

**Figure 2:**
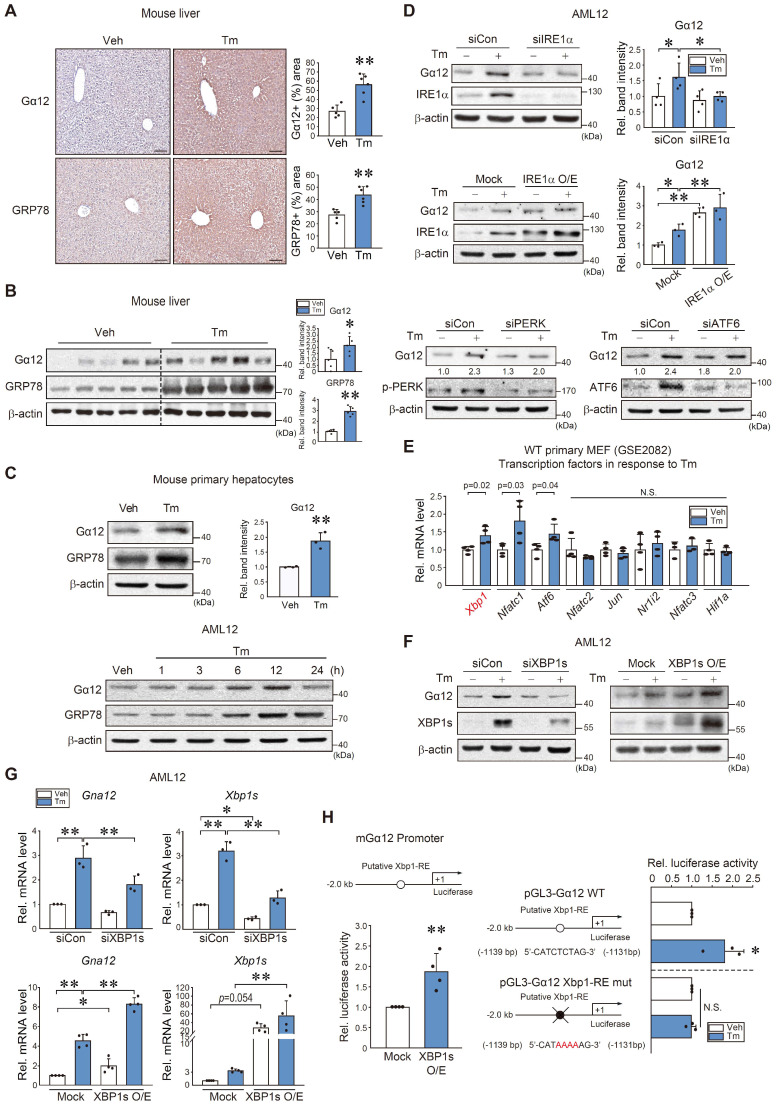
** Xbp1s-dependent *Gna12* transactivation in hepatocytes. (A)** Ιmmunohistochemistry for Gα_12_ and GRP78 in the liver of mice treated with a single dose of Tm (2 mg/kg BW, i.p., 72 h) (left). Percent areas of Gα_12_ and GRP78 were assessed using the Image J program (right*,* n = 5 or 6 each). scale bar, 200 μm. **(B)** Immunoblottings for Gα_12_ and GRP78 in the same samples as in A (left). Densitometric band intensities represent values relative to respective control (right, n = 5 each). **(C)** Immunoblottings for Gα_12_ and GRP78 in mouse primary hepatocytes treated with Tm (2 μg/ml, 12 h) (upper left) and densitometric band intensities relative to respective control (upper right, n = 3 each). Immunoblottings for Gα_12_ and GRP78 in AML12 cells were treated with Tm (2 μg/ml) for the indicated times (lower, n = 3 each; repeated 3 times with similar results). **(D)** Immunoblottings for Gα_12_ and IRE1α in AML12 cells treated with tunicamycin (Tm; 2 μg/ml, 12 h) after transfection with IRE1α siRNA (or siCon) (100 nM, 24 h) (upper) and WT-IRE1α (or mock vector) (1 μg, 24 h) (middle, n = 3 or 4 each). Immunoblottings for Gα_12_, p-PERK, and ATF6 in AML12 cells treated with Tm (2 μg/ml, 12 h) after transfection with siCon or siPERK or siATF6 (100 nM each, 24 h) (lower, n = 3 each; repeated 3 times with similar results; scan values were shown for representative blots). **(E)** Levels of major transcription factor mRNAs in primary MEF cells treated with Tm (2 μg/ml, 4 h) relative to the vehicle control derived from a publicly available dataset (GSE2082) (n = 3 or 4 each). **(F)** Immunoblotting analyses for Gα_12_ and XBP1s in AML12 cells treated with Tm (2 μg/ml, 12 h) after transfection with siXBP1s (or siCon) (100 nM, 24 h) and WT-XBP1s (or mock vector) (1 μg, 24 h) (n = 3 each). **(G)** qRT-PCR assays for *Gna12* and *Xbp1s* in AML12 cells treated as in F (n = 3 or 4 each). **(H)** *Gna12* promoter-reporter assays. Luciferase activity was measured in AML12 cells after transfection with a *Gna12* luciferase construct and a plasmid encoding XBP1s (mock or active form, 0.5 μg, 24 h) (left, n = 4 each). The cells were treated with Tm or a vehicle after transfection with an Xbp1-RE WT or Xbp1-RE mutant luciferase construct (1 μg, 24 h) (right, n = 3 each). For A-E, G, and H, the values represent the mean ± SD (*P < 0.05, **P < 0.01). Statistical significance was tested via two-tailed Student's *t*-tests or one-way ANOVA with the LSD multiple comparison procedure, where appropriate.

To understand the direct effect of ER stress on Gα_12_, we used Tm as an inducer of ER stress and found that treatment of WT mice with Tm caused an increase in Gα_12_ and GRP78 staining intensity (Figure 2A). Immunoblotting also verified increases in Gα_12_ and GRP78 levels (Figure 2B). This outcome was confirmed using mouse primary hepatocytes and AML12 cells (Figure 2C). We then explored the canonical ER stress pathways that lead to ER stress-mediated Gα_12_ induction. siRNA or overexpression approaches showed that Gα_12_ overexpression elicited by Tm primarily depends on IRE1α (Figure 2D). siIRE1α transfection alone unaffected Gα_12_ level, whereas IRE1 overexpression enhanced it.

Based on the analysis of a gene expression omnibus (GEO) dataset (GSE2082), Tm-treated primary MEF cells exhibited significant increases in *Xbp1*, *Nfatc1*, and *Atf6* mRNA levels (Figure 2E). Given that the IRE1α pathway controls the activation of Xbp1, we examined the effect of Xbp1 on Gα_12_. As expected, modulations of Xbp1 appropriately modified Gα_12_ protein and transcript levels in AML12 cells in response to Tm exposure (Figure 2F-G).

Further, a putative Xbp1 binding site was identified within the -2 kb promoter region of *Gna12* using PROMO analysis. Therefore, we then assessed whether ER stress transactivates this gene through Xbp1. Luciferase assays confirmed the binding of Xbp1 to the promoter region of *Gna12* (Figure 2H, left). In addition, Tm treatment enhanced luciferase expression using a pGL3-Gα_12_ reporter gene, but this effect was completely abolished in the assays using an Xbp1-RE mutant Gα_12_ reporter construct (Figure 2H, right). These results demonstrate that the IRE1α pathway transactivates* Gna12* through Xbp1.

### Promotion of hepatocyte ferroptosis and lipid peroxidation by Gα_12_

**Figure 3:**
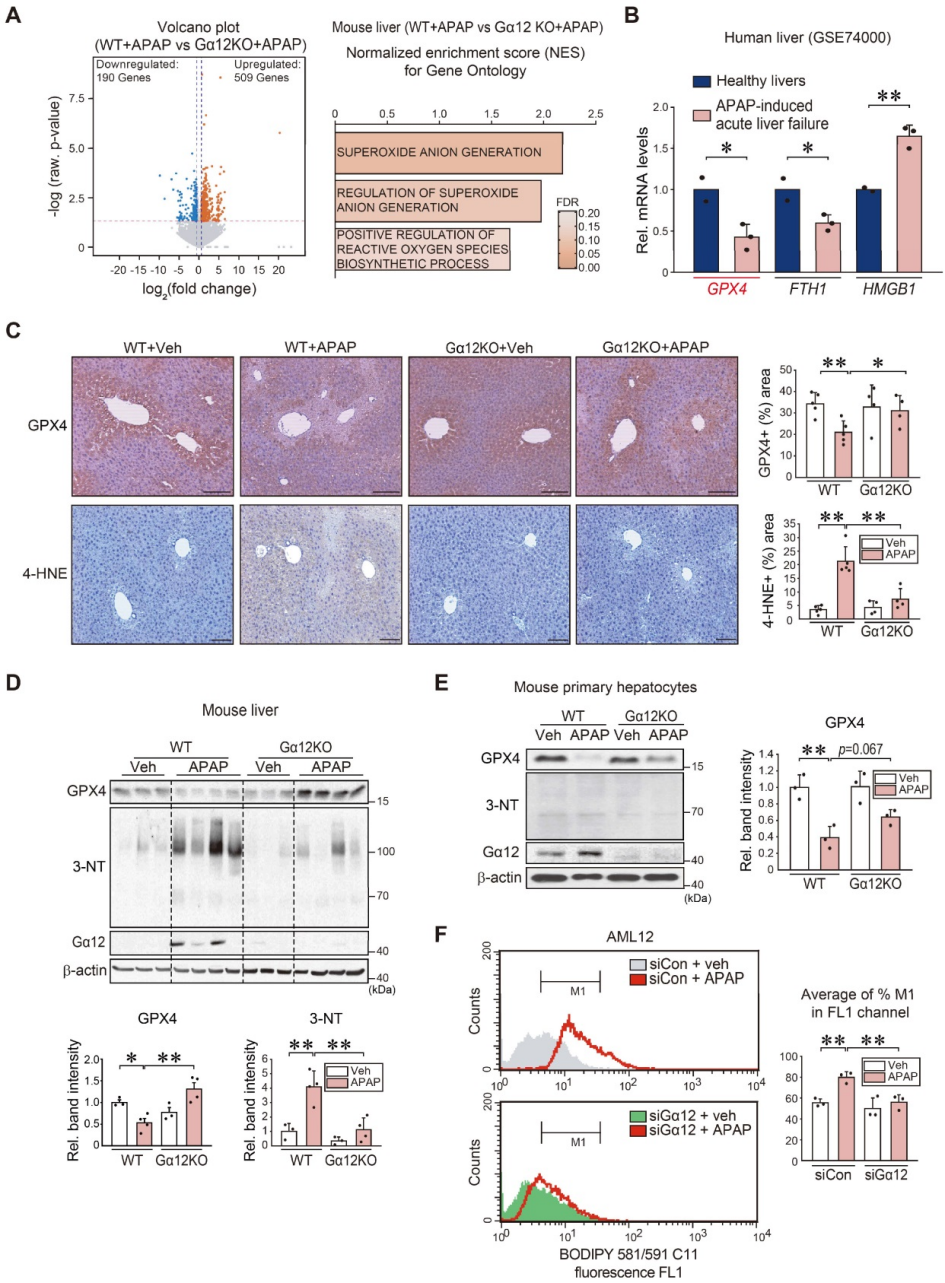
** Hepatocyte ferroptosis mediated by Gα_12_-dependent peroxidation upon APAP exposure. (A)** Volcano plot representation of differentially expressed genes between APAP-treated WT and *Gna12* KO mice using RNA-seq analysis. The horizontal and vertical lines indicate the filtering criteria (absolute fold-change ≥ 1.5 and raw *P* < 0.05). The red and blue dots indicate the upregulated (509 genes) or downregulated (190 genes) genes (left, n = 3 each). GO terms associated with ROS production were obtained between WT and *Gna12* KO mice treated with APAP (300 mg/kg BW, 6 h) (right, n = 3 each). NES and FDR are shown in the bar graph. **(B)** *GPX4*, *FTH1*, and *HMGB1* transcript levels in patients with acute liver failure. Hepatic transcripts levels were assessed using a publicly available dataset (GSE74000) from healthy individuals (normal) or patients with APAP-induced acute liver failure (n = 2 or 3 each). **(C)** Immunohistochemistry for GPX4 and 4-HNE (scale bar: 200 μm). Percent areas of GPX4 and 4-HNE staining were assessed in the livers of WT or *Gna12* KO mice treated with APAP (300 mg/kg BW, 6 h) using the Image J program (n = 4 or 5 each). **(D)** Immunoblottings for GPX4, 3-NT, and Gα_12_ in the livers of the same mice as in C. The band intensities represent values relative to their respective control (n = 3 or 4 each). **(E)** Immunoblottings for GPX4, 3-NT, and Gα_12_ in WT or *Gna12* KO primary hepatocytes treated with APAP (10 mM, 12 h). The band intensities represent values relative to the respective controls (n = 3 each). **(F)** Histograms of BODIPY 581/591 C11 fluorescence at a 530 nm emission wavelength (FL1 channel) in AML12 cells treated with vehicle or APAP (10 mM, 24 h) after transfection with siCon or siGα_12_ (100 nM each, 24 h; the experiments were simultaneously performed). Averages of M1 proportion (%) in the FL1 channel were also measured (n = 3 each). For B-F, values were expressed as mean ± SD (*P < 0.05, **P < 0.01). Statistical significance was tested via two-tailed Student's *t*-tests or one-way ANOVA coupled with Tukey's HSD or the LSD multiple comparison procedure, where appropriate.

Next, we used a gene-deficient *Gna12* KO experimental model to study whether Gα_12_ modulation affects APAP-induced liver injury (Figure S2A). To identify the mechanistic basis of this phenomenon, we analyzed an RNA-seq dataset for APAP-treated WT or *Gna12* KO mice, and found 509 upregulated genes and 190 downregulated genes (Figure 3A, left). Moreover, we conducted GSEA to examine gene ontology sets; Gene sets associated with superoxide anion generation were among the top 25 most enriched genes in APAP-treated WT mice compared to their *Gna12* KO counterparts. Gene sets associated with the 'regulation of superoxide anion generation' and 'positive regulation of reactive oxygen species biosynthetic process' pathways were also significantly enriched, being consistent with the effect of Gα_12_ overexpression on ferroptosis (Figure 3A, right). We then assessed the levels of the biomarker of ferroptosis including *GPX4*, *FTH1*, and *HMGB1* using a public GEO dataset (GSE74000) obtained from patients with APAP-induced acute liver failure. Among these genes, *GPX4* (i.e., a ferroptosis marker) transcript levels showed the biggest change (Figure 3B). We next determined whether Gα_12_ ablation prevents APAP from inducing ROS generation. The ablation of Gα_12_ substantially prevented GPX4 downregulation and also diminished the staining intensity of 4-hydroxynonenal (4-HNE) in the liver after APAP intoxication (Figure 3C), as corroborated by changes in 3-nitrotyrosine (3-NT) and GPX4 levels (Figure 3D). The higher intensities of GPX4 in Figure 3D may have resulted from slight overloading of samples and/or differences in analytical methods. The role of Gα_12_ in ferroptosis was validated using primary hepatocytes (Figure 3E) or AML12 cells (Figure S2B). The impact of Gα_12_ modulation on lipid peroxidation was further determined in the cells treated with APAP using the BODIPY 581/591 C11 oxidation method. Transfection with siRNA targeting Gα_12_ caused a significant shift in cell populations in response to APAP treatment (Figure 3F).

DL-buthionine-[S, R]-sulfoximine (BSO) leads to inhibit GSH synthesis with reduced GPX4 activity, thus promoting ferroptosis 16. Therefore, we employed an *in vivo* BSO treatment model and identified Gα_12_ overexpression. Consistently, GPX4 was markedly repressed, as demonstrated by immunohistochemistry, which coincided with the liver injury area (Figure S3A). This outcome was confirmed using immunoblotting (Figure S3B). Additionally, we assessed the sulfhydryl-depleting effect of BSO on Gα_12_ and GPX4 levels in cells (Figure S3C, left). Similar results were obtained when we used Erastin (Figure S3C, right). To assess the causal relationship between Gα_12_ and ferroptosis, we further examined whether treatment with ferrostatin-1 (Fer-1), a specific inhibitor of ferroptosis 17, changes Gα_12_ expression in mice, and observed that Fer-1 treatment reversed the effects of APAP on Gα_12_ and GPX4 with almost a complete normalization of blood liver injury markers (Figure S4A-C). This effect was verified in cells treated with either BSO or Erastin (Figure S4D). Thus, it is highly likely that ferroptosis induces Gα_12_ expression. Together, these results provide strong evidence that the ER stress-mediated induction of Gα_12_ contributes to ferroptosis, which further amplifies Gα_12_ overexpression in a cyclical fashion, thus worsening hepatocyte injury.

### Gα_12_-mediated ALOX12 induction in APAP-intoxicated livers

**Figure 4:**
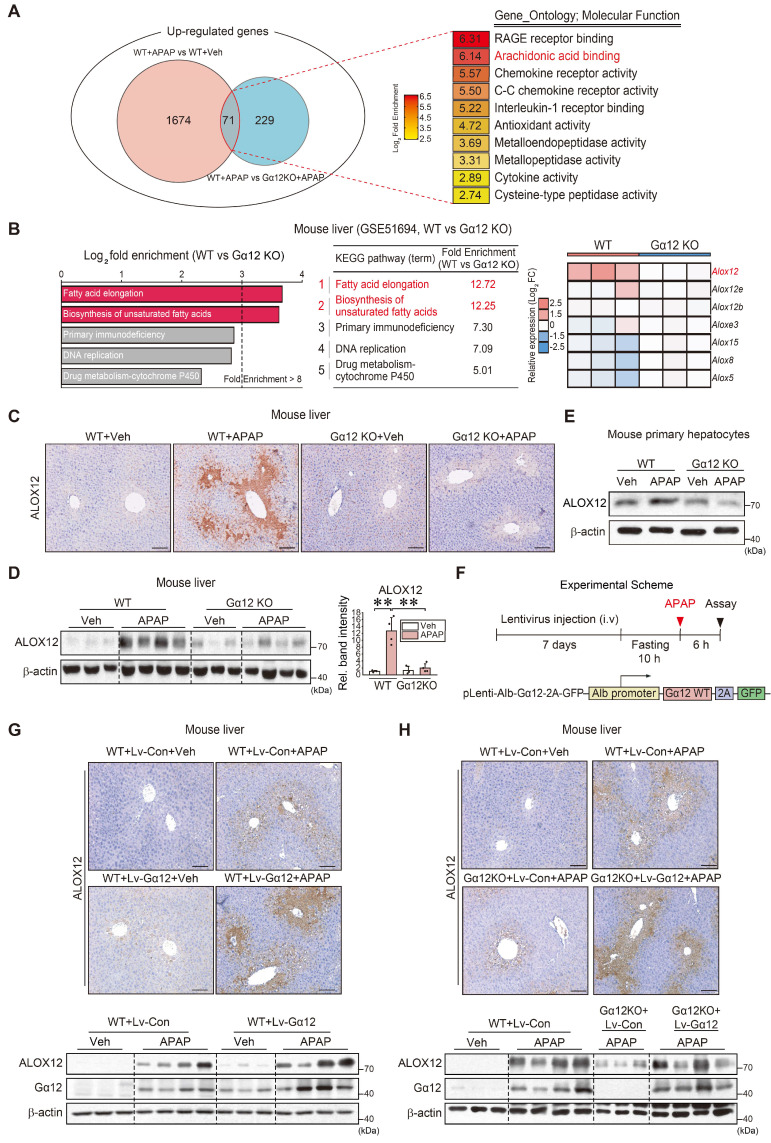
** Gα_12_-mediated induction of ALOX12 by APAP intoxication. (A)** Venn diagrams showing the overlap of commonly upregulated genes in the liver of mice treated with APAP (300 mg/kg BW, 6 h) versus vehicle treatment (fold change ≥ 2.5 and raw *P* < 0.05) and WT mice treated with APAP (300 mg/kg BW, 6 h) versus *Gna12* KO mice treated with APAP (fold change ≥ 2 and raw *P* < 0.05) (left, n = 3 each). GO term enrichment (molecular function) of the genes highlighted for functions such as arachidonic acid binding (second rank) (right). **(B)** KEGG pathway analysis using a cDNA microarray dataset (GSE51694) obtained from the liver of WT and *Gna12* KO mice. Ferroptosis-related pathways are indicated in red (left and middle). Heatmap of *Alox* isoform transcript levels in the livers of WT and *Gna12* KO mice (right, n = 3 each). **(C)** Immunohistochemistry for ALOX12 in the livers of WT or *Gna12* KO mice treated with APAP (300 mg/kg BW, 6 h) (n = 3 or 4 each). **(D)** Immunoblotting for ALOX12 in the liver of WT or *Gna12* KO mice treated with APAP (300 mg/kg BW, 6 h). The densitometric band intensities represent values relative to the respective control (n = 3 or 4 each). **(E)** Immunoblotting for ALOX12 in WT or *Gna12* KO primary hepatocytes treated with APAP (10 mM, 12 h) (n = 3). **(F)** A schematic showing a construct encoding Gα_12_-WT (Lv-Alb-Gα_12_) downstream from the albumin promoter. The mice were injected with a single dose of albumin promoter-Gα_12_-WT lentivirus (or Lv-control) through the tail vein. After one week, the mice were fasted overnight prior to APAP treatment and sacrificed 6 h thereafter. **(G)** Representative immunohistochemistry (upper) and immunoblottings (lower) for ALOX12 and Gα_12_ in WT mice treated with APAP (300 mg/kg BW, 6 h) one week after injection with Lv-con or Lv-Gα_12_ via the tail vein (n = 3 or 4 each). **(H)** Representative immunohistochemistry for ALOX12 (upper) and immunoblottings (lower) for ALOX12 and Gα_12_ in WT mice and *Gna12* KO mice treated with APAP (300 mg/kg BW, 6 h) one week after injection with Lv-con or Lv-Gα_12_ via the tail vein (n = 4-6 each). For D, values were expressed as mean ± SD (**P < 0.01). Statistical significance was tested via one-way ANOVA coupled with Bonferroni's method, where appropriate. scale bar: 200 μm.

Having identified the impact of Gα_12_ on ferroptosis, we then sought to elucidate the regulatory molecules downstream from Gα_12_. Therefore, to discover the shared molecular functions required for the regulation of ferroptosis, we focused on the upregulated overlapping genes between APAP treatment and vehicle treatment in WT mice, as well as APAP-treated WT mice and APAP-treated *Gna12* KO mice, using RNA-seq data. GO term enrichment analysis (molecular function) was performed on 71 genes upregulated in both groups (Figure 4A). We also conducted a KEGG pathway analysis using a *Gna12* KO liver tissue cDNA microarray dataset (GSE51694). Several biological processes including “fatty acid elongation” (the foremost affected pathway) and “biosynthesis of unsaturated fatty acids” (the second-most affected pathway) were highly enriched (Figure 4B, left). These pathways are closely associated with ferroptosis, as polyunsaturated fatty acids (PUFAs) in lipid membranes react with ROS 18. Among these molecules, we focused on lipoxygenases because they belong to a family of nonheme, iron-containing enzymes catalyzing the deoxygenation of PUFAs. In the analysis of cDNA microarray datasets obtained from WT and *Gna12* KO livers, *Alox12* was the most highly upregulated among major isoforms (Figure 4B, right).

As expected, APAP treatment upregulated ALOX12 in WT mice. A reversal of ALOX12 expression by a Gα_12_ deficiency was confirmed in primary hepatocytes (Figure 4C-E). To confirm the effects of liver-specific Gα_12_ overexpression on ALOX12 with more precision, we utilized a lentiviral albumin-Gα_12_ (Lv-Alb- Gα_12_) delivery system in WT or *Gna12* KO animals subjected to APAP challenge (Figure 4F). We then analyzed the protein levels in the liver of WT and *Gna12* KO mice subjected to hepatocyte-specific overexpression of Gα_12_. The overexpression of Gα_12_ enhanced the ability of APAP to induce ALOX12 (Figure 4G). Moreover, the lack of ALOX12 induction in *Gna12* KO mice was reversed by Gα_12_ overexpression (Figure 4H and S5A). In Figure S5B, the increasing effect of APAP on the *Alox12* transcript was statistically non-significant, but the fold-changes in the absence of Gα_12_ seemed to be attenuated. Also, hepatocyte-specific Gα_12_ overexpression in *Gna12* KO mice repressed GPX4 (Figure S5C). These results demonstrate that Gα_12_ overexpression enhances the effects of APAP on ALOX12 and GPX4, which leads to peroxide production and ultimately results in hepatocyte ferroptosis.

### Induction of ALOX12 as mediated by miR-15a dysregulation

**Figure 5:**
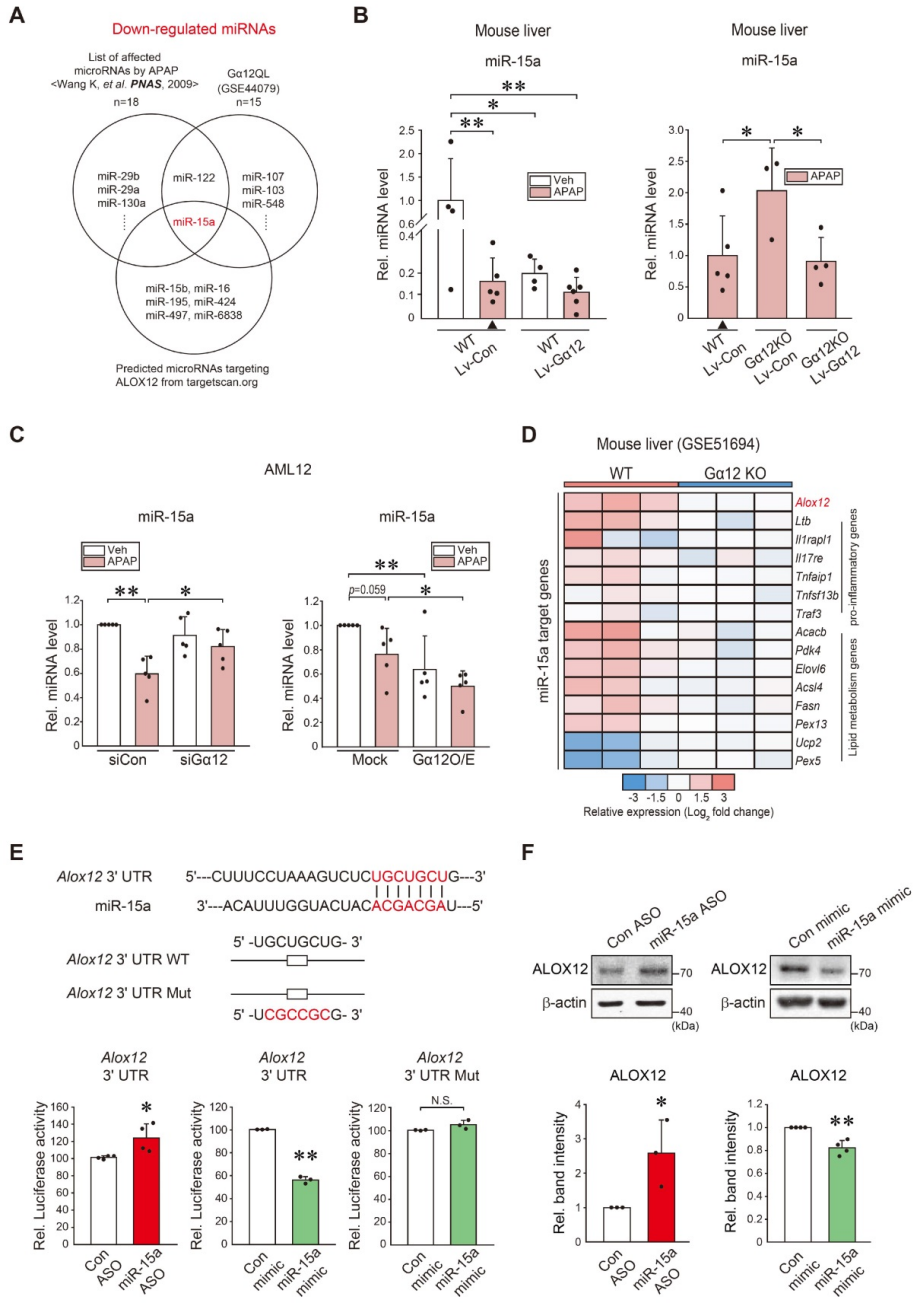
** Identification of miR-15a as an inhibitor of ALOX12 downstream from Gα_12._ (A)** Venn diagrams showing miR-15a expression among the downregulated miRNAs in the liver of mice treated with APAP (300 mg/kg, 24 h) (Wang K, et al. *PNAS,* 2009), Huh7 cells with Gα_12_QL transfection (GSE44079), and those predicted to inhibit ALOX12 based on our Target Scan database analyses; 18 miRNAs were downregulated in the first set, whereas a total of 15 downregulated miRNAs were identified in the second set. Seven miRNAs were predicted to target ALOX12. **(B)** qRT-PCR assays for miR-15a using the same mice as in Fig. 4G (n = 4-6 each) or Fig. 4H (n = 3-5 each). Experiments were done at the same time and the marked control group (▲) was shared for statistical analysis. **(C)** qRT-PCR assays for miR-15a in AML12 cells treated with APAP (10 mM, 12 h) after transfection with siGα_12_ (or siCon) (100 nM, 24 h, n = 5 each) (left), or the plasmid encoding for Gα_12_ or a mock vector (1 μg, 24 h, n = 5 each) (right). **(D)** Heatmap of differentially expressed miR-15a target genes based on the GSE51694 dataset from the livers of WT and *Gna12* KO mice; *Alox12,* 6 pro-inflammatory genes, and 8 lipid metabolism genes (n = 3 each). **(E)** Prediction of miR-15a binding to the 3'-UTR of Alox12 mRNA (upper). *Alox12*-3'-UTR luciferase assays in AML12 cells transfected with miR-15a ASO (or control ASO) (100 nM, 48 h), or miR-15a mimic (or control mimic) (100 nM, 24 h) (lower, n = 3 or 4 each). **(F)** Immunoblottings for ALOX12 in AML12 cells transfected with miR-15a ASO (or control ASO) (100 nM, 48 h), or miR-15a mimic (or control mimic) (upper, 100 nM, 24 h). The densitometric band intensities represent values relative to the respective control (lower, n = 3 or 4 each). For B, C, E, and F, values were expressed as mean ± SD (*P < 0.05, **P < 0.01). Statistical significance was tested via two-tailed Student's *t*-tests or one-way ANOVA with the LSD multiple comparison procedure, where appropriate.

Given that Gα_12_ deficiencies led to minor changes in *Alox12* transcripts relative to its protein levels, we next explored microRNA-dependent post-transcriptional processes. Candidates were predicted using the Target Scan program after analyzing the overlap between microarray datasets from Gα_12_QL (an active mutant form)-Huh7 cells (GSE44079), miRNAs downregulated by APAP intoxication in mice 19, and those putatively bound to the 3'-UTR of ALOX12 mRNA (Figure 5A). Among these, we selected microRNA (miR)-15a as a potential inhibitor of ALOX12. As expected, APAP treatment significantly and substantially diminished miR-15a levels. Specifically, the lentiviral delivery of Gα_12_ to hepatocytes *in vivo* suppressed the basal miR-15a expression. Combining APAP treatment and Gα_12_ overexpression in hepatocytes tended to further decrease miR-15a levels (Figure 5B, left). Moreover, ablation of Gα_12_ lessened the ability of APAP to inhibit miR-15a, which was reversed by hepatocyte-specific delivery of Gα_12_ (Figure 5B, right). Similarly, silencing of Gα_12_ increased miR-15a level in response to APAP treatment (Figure 5C, left). The opposite outcome was obtained in cells with Gα_12_ overexpression (Figure 5C, right). MiR-15a may regulate other targets such as pro-inflammatory (6 genes) and lipid metabolism (8 genes) pathways. Among these, *Alox12* displayed a higher fold change in the liver of WT mice compared to *Gna12* KO mice (Figure 5D).

Next, we assessed the effect of miR-15a on ALOX12 using an *Alox12* 3'-UTR reporter construct. There was a practically complete pairing between *Alox12* 3'-UTR and the miR-15a seed sequence (Figure 5E, upper). Transfection with miR-15a antisense oligonucleotide (ASO) and miR-15a mimic significantly altered *Alox12* 3'-UTR luciferase activity and miR-15a mimic transfection effect was not observed in the assay using an *Alox12* 3'-UTR mutant reporter (Figure 5E, lower). The inhibitory effect of miR-15a on ALOX12 was strengthened by the outcomes of experiments using an ASO inhibitor or mimic (Figure 5F). Collectively, these data show that miR-15a inhibits ALOX12 in hepatocytes, which may be dysregulated by APAP intoxication downstream from Gα_12_.

### Prevention of ALOX12 induction and ferroptosis by ROCK1 inhibition

**Figure 6:**
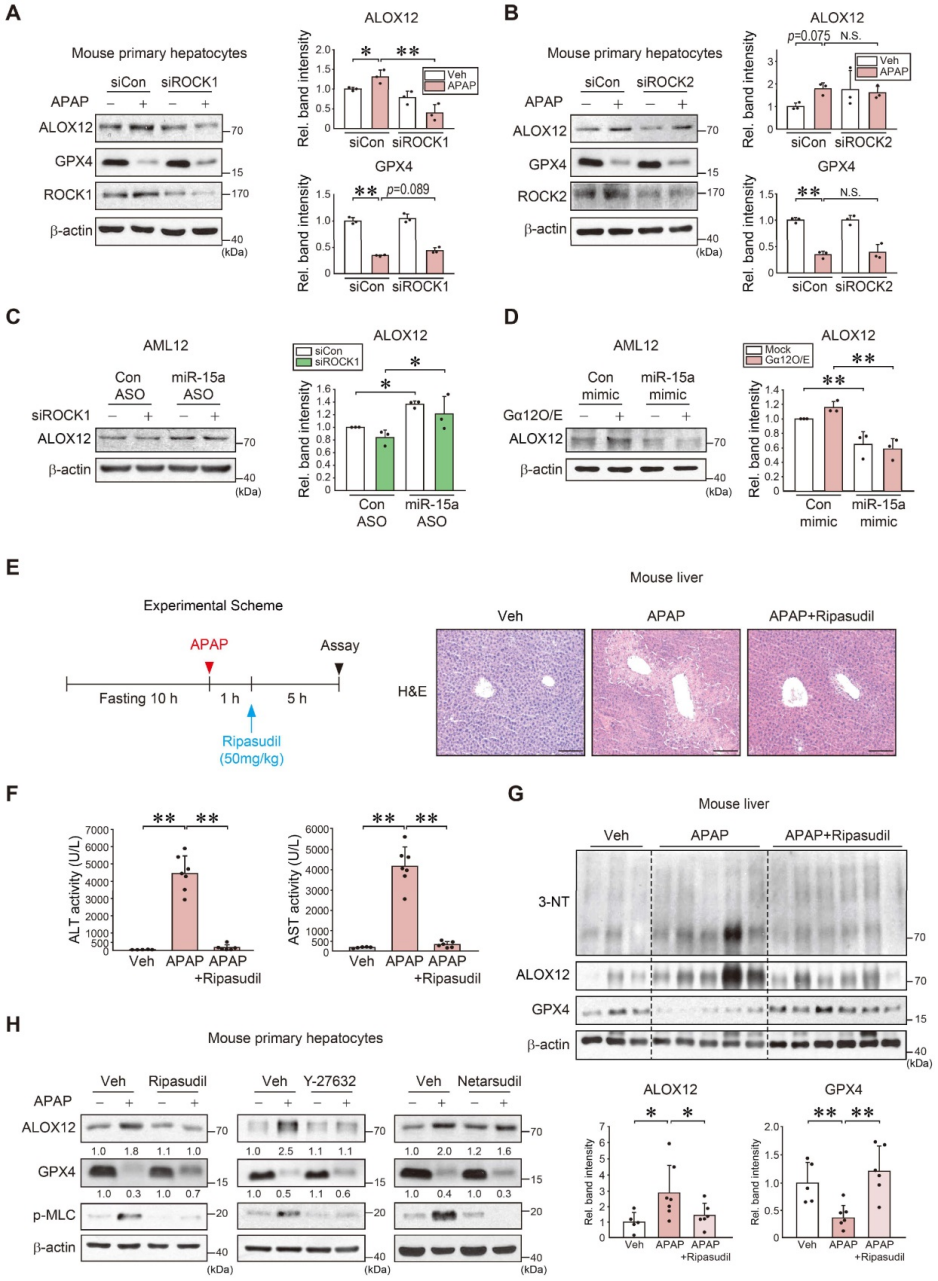
** Inhibition of APAP-induced ferroptosis by ROCK1 inhibitors. (A)** Immunoblottings for ALOX12, GPX4, and ROCK1 in mouse primary hepatocytes treated with APAP (10 mM, 12 h) after transfection with siROCK1 (or siCon) (100 nM, 24 h). **(B)** Immunoblottings for ALOX12, GPX4, and ROCK2 in mouse primary hepatocytes treated with APAP (10 mM, 12 h) after transfection with siROCK2 (or siCon) (100 nM, 24 h). **(C)** Immunoblotting of ALOX12 in AML12 cells transfected with miR-15a ASO (or control ASO) (100 nM, 48 h) after transfection with siROCK1 (100 nM, 24 h). **(D)** Immunoblotting of ALOX12 in AML12 cells transfected with miR-15a mimic (or control mimic) (100 nM, 24 h) after transfection with Gα_12_ (1 μg, 24 h). **(E)** A schematic showing APAP and ripasudil treatment (left). Liver histopathology in WT mice treated with ripasudil (50 mg/kg BW, 5 h) 1 h after APAP treatment (300 mg/kg BW, 6 h) (right, n = 5-7 each). scale bar: 100 μm. **(F)** Serum ALT and AST activities in the same mice as in E. **(G)** Immunoblottings for 3-NT, ALOX12, and GPX4 in the same mice as in E. **(H)** Immunoblotting analyses for ALOX12, GPX4, and p-MLC in WT primary hepatocytes treated with APAP (10 mM, 12 h) 1 h after treatment with ripasudil (50 μM), Y-27632 (10 μM), or netarsudil (2 μM) (n = 3 each). For A-D and G, the band intensities represent values relative to the respective control (n = 3-6 each). For A-D, F, and G, values were expressed as mean ± SD (*P < 0.05, **P < 0.01). Statistical significance was tested via one-way ANOVA coupled with Tukey's HSD or the LSD multiple comparison procedure, where appropriate.

ROCK1/2 are key effectors of the Gα_12_-RhoA pathway 20. Given the association between Gα_12_, the RhoA-ROCK pathway, and ferroptosis, our findings indicated that miR-15a acts as an ALOX12 inhibitor, we next examined the role of each ROCK isoform and their pharmacological inhibition. First, siRNA knockdown experiments were conducted to confirm the selectivity of ROCK1 in APAP-induced ferroptosis. Knockdown of ROCK1, but not ROCK2, prevented ALOX12 induction. ROCK1 silencing resulted in a moderate GPX4 recovery in primary hepatocytes and AML12 cells (Figure 6A-B and S6A). ALOX12 inhibition by ROCK1 knockdown was reversed by miR-15a ASO transfection (Figure 6C). Lastly, we confirmed the inhibitory effect of miR-15a mimic on the induction of ALOX12 via Gα_12_ overexpression (Figure 6D). Next, we assessed the effect of ripasudil (a ROCK1 preferential inhibitor) treatment on WT mice after a single dose of APAP (Figure 6E). Interestingly, this compound was found to completely inhibit the onset of acute liver injury. The ability of ripasudil to rescue APAP intoxication was confirmed by ALT and AST assays (Figure 6F). Lipid peroxidation was also suppressed and ALOX12 induction was attenuated with increased GPX4 levels (Figure 6G). Therefore, ripasudil treatment was deemed an effective means to inhibit APAP-induced ferroptosis.

Moreover, treatment with ripasudil inhibited ALOX12 and led to a recovery of GPX4 levels. Y-27632 also decreased ALOX12, albeit with an insignificant GPX4 recovery. In contrast, netarsudil (a selective ROCK2 inhibitor) rendered minimal effects. The levels of p-MLC, a downstream marker of ROCK, were similarly affected (Figure 6H and S6B). These results demonstrate that pharmacological inhibitors or ROCK1-specific siRNA prevent APAP-induced ferroptosis.

### Amelioration of APAP-induced liver injury and inflammation by Gα_12_ ablation

**Figure 7:**
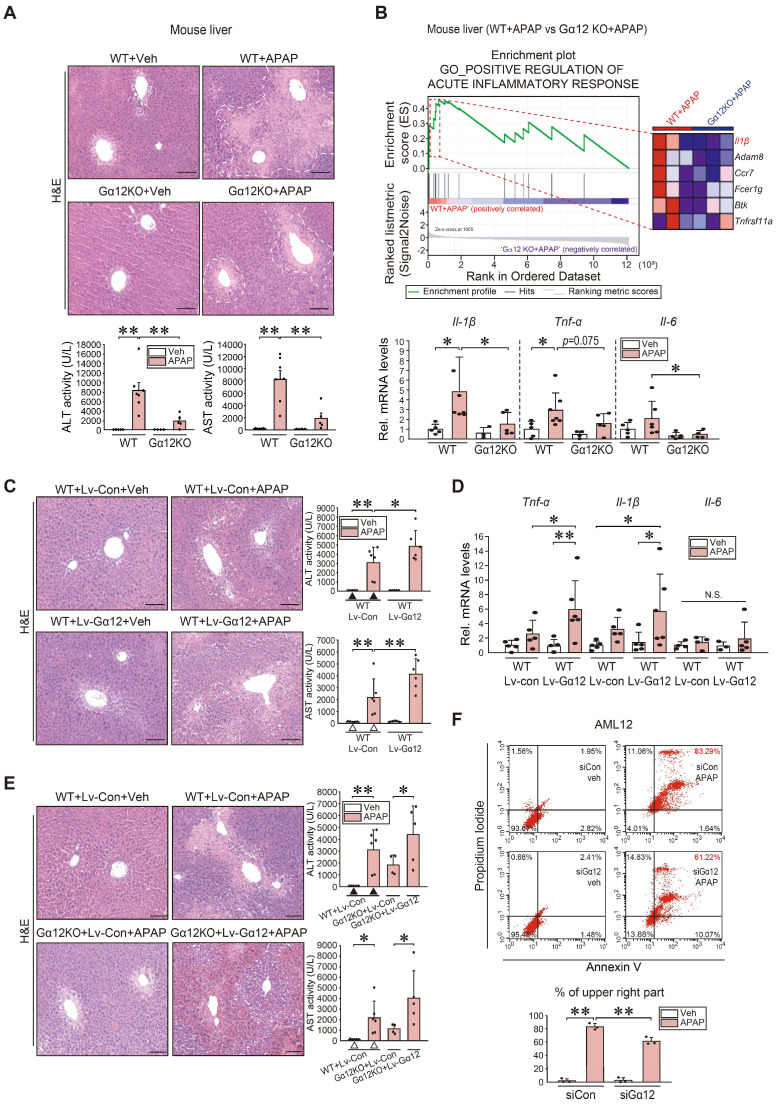
** Increase in inflammatory injuries of hepatocytes by Gα_12_ overexpression. (A)** Liver histopathology (H&E), serum alanine transaminase (ALT), and serum aspartate transaminase (AST) activities in WT or *Gna12* KO mice treated with APAP (300 mg/kg BW, 6 h) (n = 4-7 each). **(B)** GSEA plot of GO categories, leading-edge genes (upper, n = 3 each) (NES = 1.94, FDR < 0.1), and qRT-PCR assays in the same mice as in A (lower, n = 3-7 each). **(C)** Liver histopathology, ALT, and AST activities in the same mice as in Fig. 4G (n = 5 or 6 each). **(D)** qRT-PCR assays for inflammatory cytokines in the same mice as in Fig. 4G (n = 3-6 each). **(E)** Liver histopathology (left), ALT and AST activities (right) in the same mice as in Fig. 4H. For C and E, experiments were done at the same time and the marked control groups (▲ and △) were respectively shared for statistical analyses. **(F)** Flow cytometric analyses for fluorescein isothiocyanate-annexin V and propidium iodide (upper). The average proportion (%) of the upper right portion was measured (lower). AML12 cells were treated with APAP (10 mM, 24 h) after transfection with siCon or siGα_12_ (100 nM each, 24 h) (n = 3 each). For A-F, values represent the mean ± SD (*P < 0.05, **P < 0.01). Statistical significance was tested via one-way ANOVA coupled with Tukey's HSD or the LSD multiple comparison procedure, where appropriate. scale bar: 100 μm.

To understand the functional impact of Gα_12_ modulations on APAP intoxication more in depth, we employed Gα_12_ genetic knockout and hepatocyte-specific overexpression techniques using a mouse model. Histopathological analyses demonstrated that Gα_12_ ablation substantially ameliorated liver injury elicited by a single APAP dose. This effect was confirmed by the results of blood biochemical assays (Figure 7A). GSEA was then performed using RNA-seq data obtained from WT and *Gna12* KO mice treated with APAP to assess how Gα_12_ affects liver damage. Interestingly, most of the elucidated liver injury-related pathways were linked to the inflammatory response. To understand the functional significance of this pathway, we examined the function of the leading edge genes (red-dotted box) upregulated in APAP-treated WT compared to APAP-treated *Gna12* KO mice. Notably, we identified *Il-1β* as a key molecule in the 'positive regulation of acute inflammatory response' GO pathway (Figure 7B, upper and S7A). APAP-treated WT mice exhibited significant increases in the transcript levels of *Il-1β*, *Tnf-α*, and *Il-6* in the liver, which was significantly attenuated by *Gna12* KO. However, the *Gna12* KO effect was not seen in the assays for anti-inflammatory biomarkers including *Arg1*, *Cd206,* and *Ym1* (Figure 7B, lower and S7B).

To verify the effects of liver-specific Gα_12_ overexpression, we employed an Lv-Alb-Gα_12_ system in WT or *Gna12* KO animals subjected to APAP challenge. As expected, more severe hepatic injuries and inflammatory responses were observed in WT mice due to hepatocyte-specific overexpression of Gα_12_ (Figure 7C-D and S7C). Similarly, the liver-specific Gα_12_ delivery to *Gna12* KO mice abrogated the liver-protective effect (Figure 7E). In contrast, Gα_12_ deficiency significantly decreased necrotic cell populations. Moreover, Gα_12_ siRNA transfection decreased the population of propidium iodide (PI) and Annexin V double-positives in cells treated with APAP (Figure 7F). Likewise, APAP-induced increases in the number of TUNEL-positive cells in the liver were significantly diminished in *Gna12* KO mice (Figure S7D). In addition, adenoviral delivery of Gα_12_ to AML12 cells enhanced the ability of Tm to increase Annexin V/PI double-positive cell populations (Figure S7E). Together, these findings strongly support the role of ER stress-mediated Gα_12_ overexpression in hepatic inflammation and necrosis in response to APAP intoxication.

### Dysregulation of Gα_12_, ALOX12, and miR-15a in patients with acute liver injury or fibrosis/cirrhosis

**Figure 8:**
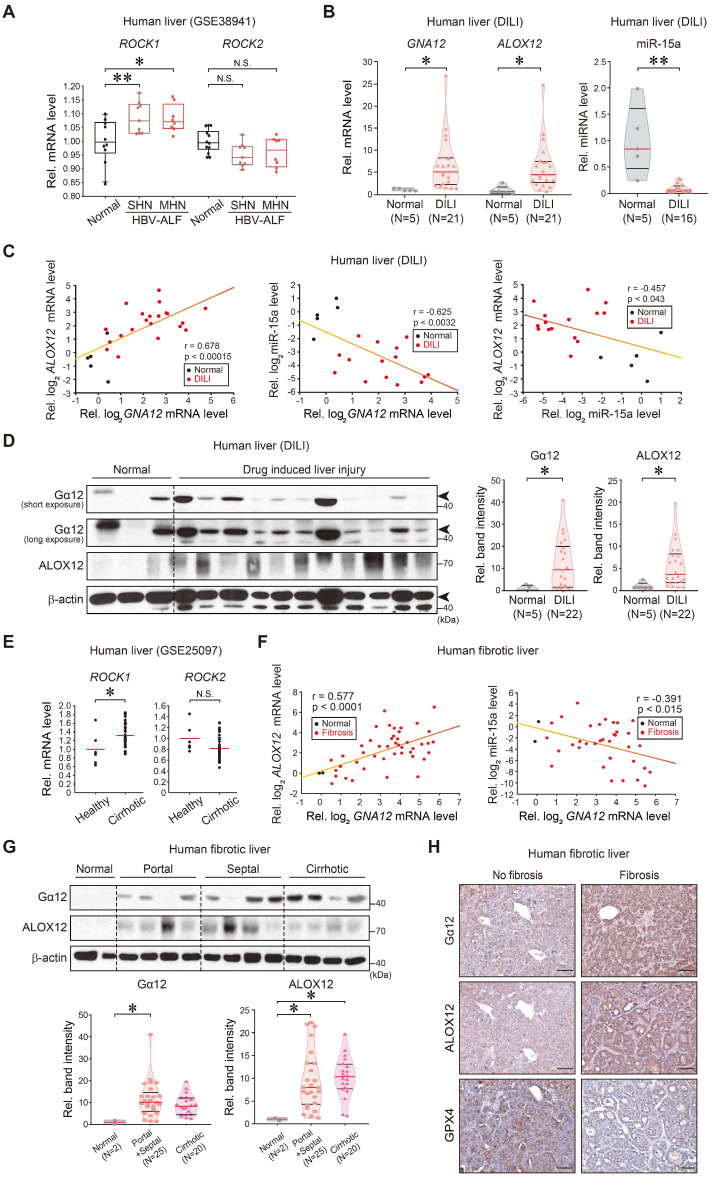
** Correlations between Gα_12_-ROCK1, ALOX12, miR-15a, and GPX4 in liver specimens of patients with acute liver injury or liver fibrosis. (A)** *ROCK1* and *ROCK2* transcript levels in the livers of healthy individuals or patients with HBV-acute liver failure (ALF). MHN, massive hepatic necrosis; SHN, submassive hepatic necrosis. The data are reported as a box-and-whisker plot. Box, interquartile range (IQR); whiskers, 5-95 percentiles; horizontal line within the box, median (n = 10 samples from 10 individual normal subjects; n = 8 samples from 2 patients with SHN-ALF; n = 9 samples from 2 patients with MHN-ALF; as described in the GSE38941 database). **(B)** *GNA12*, *ALOX12*, and miR-15a transcript levels in the livers of healthy individuals (n = 5) or patients with DILI (n = 21 for *GNA12* and *ALOX12*, and n = 16 for mir-15a). In some of the patient samples, *GNA12* (n = 1), *ALOX12* (n = 1) and miR-15a (n = 6) levels were undetectable. **(C)** Correlations between *GNA12* and *ALOX12* (left, n = 26), or *GNA12* and miR-15a (middle, n = 20), or miR-15a and *ALOX12* (right, n = 20) transcripts. In some samples, *GNA12* (n = 1), *ALOX12* (n = 1) and miR-15a (n = 6) levels were undetectable. **(D)** Immunoblottings for Gα_12_ and ALOX12 in healthy individuals (n = 5) or patients with DILI (left, n = 22), and their quantifications (right, n = 27). Representative blots were shown. Short and long exposures were shown for comparison. **(E)** *ROCK1* and *ROCK2* transcript levels in the livers of healthy individuals or a large cohort of patients with liver fibrosis (GSE25097, n = 46). **(F)** Correlations between *GNA12* and *ALOX12* (left, n = 47), or *GNA12* and miR-15a (right, n = 38) transcripts in patients with no fibrosis and liver fibrosis. MiR-15a levels in 9 patients with fibrosis were undetectable. **(G)** Immunoblottings for Gα_12_ and ALOX12 in patients with no fibrosis (Metavir score F0) or patients with the portal (Metavir score F1, 2) or septal (Metavir score F3) fibrosis, or those with cirrhotic (Metavir score F4) lesion (upper), and their quantifications (lower, n = 47). Fibrosis was scored as F0 (absent), F1 (portal fibrosis), F2 (portal fibrosis with few septa), F3 (septal fibrosis), and F4 (cirrhosis) according to the Metavir Score System. **(H)** Immunohistochemistry for Gα_12_, ALOX12, and GPX4 in patients without fibrosis (n = 2) or septal fibrosis (right, n = 10). Representative liver sections are shown. scale bar: 200 μm. For A and E, Values were expressed as mean ± SD (*P < 0.05, **P < 0.01). For B, D, and G, violin plots show all independent biological replicates with the median as a red straight line and the upper/lower quartiles as black straight lines (*P < 0.05, **P < 0.01). Statistical significance was tested via two-tailed Student's *t*-tests, Pearson correlation, or one-way ANOVA coupled with Tukey's HSD or the LSD multiple comparison procedure, where appropriate.

Finally, we analyzed the identified regulatory molecules in patient liver samples. Analysis of public GEO databases obtained from patients with HBV-associated acute liver failure (HBV-ALF) (GSE38941) indicated that hepatic *ROCK1* transcript levels were higher in patients with submassive hepatic necrosis (SHN) or massive hepatic necrosis (MHN) compared to healthy individuals, whereas *ROCK2* mRNA levels were not significantly affected (Figure 8A). To further assess the association of *GNA12*, *ALOX12*, and miR-15a transcript levels and liver injury in clinical situations, we analyzed samples of patients with acute liver injury on drugs. As expected, the intoxicated patients, which consisted of drugs, herb medications, and unknown origin, exhibited increases in hepatotoxicity-related values compared to normal subjects (Supplementary Table 1). Patients with acute liver injury showed significantly higher *GNA12*, *ALOX12* transcript levels along with the decrease of miR-15a as compared to healthy subjects (Figure 8B). Hepatic *GNA12* and *ALOX12* mRNA levels positively correlated with each other. The negative correlations existed between miR-15a and *GNA12*, or miR-15a and *ALOX12* (Figure 8C). Immunoblottings confirmed strong increases in Gα_12_ and ALOX12 levels in patients with acute liver injury (Figure 8D).

To extend our findings to patients with liver fibrosis, we further analyzed a public cirrhosis dataset and experimentally the non-tumorous regions of HCC patients with fibrosis; cirrhotic patients exhibited significantly higher transcript levels of *ROCK1*, but not* ROCK2* than healthy individuals in a human cirrhotic liver cohort database (GSE25097) (Figure 8E). When we analyzed liver samples of HCC patients with fibrosis, we found a positive correlation between *GNA12* and *ALOX12* mRNAs (Figure 8F, left), and an inverse correlation between miR-15a and *GNA12* mRNAs (Figure 8F, right). Moreover, we corroborated an overall increase in Gα_12_ and ALOX12 levels in the analyses of the portal, septal fibrosis, and cirrhosis subgroups divided according to fibrosis severity (Figure 8G). Among these, the septal fibrosis subgroup appeared to display the highest fold change in Gα_12_ and ALOX12 compared to the remaining subgroups. Immunohistochemistry confirmed the induction of Gα_12_ and ALOX12 with reciprocal decreases in GPX4 (Figure 8H). Taken together, these results indicate that Gα_12_ overexpression mediated by ER stress promotes ALOX12 induction in hepatocytes through ROCK1 by dysregulating miR-15a, which then promotes ferroptosis.

## Discussion

Our findings shown here indicated that ER stress-mediated Gα_12_ overexpression facilitates APAP-induced liver injury, thus elucidating a novel role of Gα_12_ in the signaling pathway culminating in acute hepatic injury. This hypothesis was further supported by the fact that Gα_12_ ablation prevented liver injury, an effect that was abrogated by hepatocyte-specific overexpression of Gα_12_. Moreover, other toxicant challenges also lead to Gα_12_ overexpression, resulting in hepatocyte ferroptosis, all of which provide novel insights into the mechanisms of liver pathology. Also, the effects of other ER stress-inducers strengthen the role of the Gα_12_ signaling axis in acute liver injury. Previously, we investigated the regulatory role of Gα_12_ in hepatic lipid metabolism for mitochondrial energy expenditure through SIRT1 and PPARα, identifying Gα_12_ as a physiological regulator that contributes to mitochondrial oxidation. However, overexpression of Gα_12_ in conjunction with ER stress unexpectedly causes hepatocyte ferroptosis presumably due to dysregulation of the coordinated functions of mitochondria and ER capacity. Thus, our findings confirm that Gα_12_ and its downstream molecules may be promising targets for the treatment of acute liver injury accompanying ER stress.

In this study, we elaborated on the role of the IRE1α-Xbp1 pathway in the transcriptional induction of *Gna12*. This, in turn, results in an ER stress-induced vicious cycle in hepatocytes, suggesting that ER stress induction of Gα_12_ may lead to hepatocyte dysfunction if not treated promptly, contributing to the development of liver injurious symptoms and subsequently fibrosis progression. Previously, we reported the role of the JNK pathway in macrophages downstream of Gα_12_ contributes to NF-κB-mediated inflammatory responses. Our study also showed augmented increases of *Il-1β*, *Tnf-α*, and *Il-6* in the liver of APAP-treated WT or Gα_12_ KO mice subjected to hepatocyte-specific overexpression of Gα_12_. Therefore, Gα_12_ overexpression in hepatocytes coupled with inflammatory cytokine production from macrophages likely leads to hepatocyte death, exacerbating injurious symptoms, as further corroborated by our analyses of human liver samples.

APAP-induced liver toxicity mainly results from apoptotic and necrotic cell death, as supported by the resulting mitochondrial permeability transition pore opening, membrane swelling, and lysis 21. Our results showing changes in AnnexinV/PI and TUNEL staining after modulation of Gα_12_ in the present study supports this idea, being also consistent with Gα_12_-JNK-dependent apoptosis. Thus, both apoptosis and necroptosis are responsible for APAP-induced hepatotoxicity 22. However, it is now accepted that apoptosis plays a limited role due to the ineffectiveness of pan-caspase inhibitors in a rodent model of APAP-induced hepatotoxicity 23. In addition, RIPK3 and MLKL were found to be dispensable 24, suggesting that necroptosis may not account for the entire necrotic cell death. An important finding of our study is that Gα_12_ plays a critical role in the ferroptosis process. NAPQI, a reactive intermediate produced from APAP metabolism, causes GSH depletion in hepatocytes, which would contribute to ferroptosis. These findings were confirmed by our experiments, indicating that Fer-1 prevents BSO- or Erastin-induced dysregulation of GPX4. While we were finishing this research, a report came out showing Fer-1 inhibition of APAP toxicity 25. Altogether, our findings elucidate previously uncharacterized features of ferroptosis and its contribution to the progression of acute liver injury.

Lipoxygenase (LOXs) catalyzed lipid peroxides production. PUFAs are the preferred substrates of LOXs and thus lead to the acceleration of the pro-ferroptotic oxidation process 26. Specifically, ALOX12 is induced in hepatocytes during ischemia-reperfusion and catalyzes the dioxygenation of PUFAs. Moreover, an increase in ALOX12 expression may facilitate a variety of stress conditions 27. Therefore, the induction of ALOX12 is likely to accelerate an inflammatory response. ALOX12 also contributes to p53-mediated ferroptosis 28. Our data clearly demonstrate that ALOX12 expression relies on Gα_12_ signaling. This was particularly demonstrated by our experiments involving hepatocyte-specific Gα_12_ gene delivery in *Gna12* KO mice. These findings reiterate the initiating role of APAP-induced Gα_12_ overexpression in the context of ALOX12 induction, elucidating new molecular mechanisms of liver pathogenesis, as well as the role of ER stress in hepatocytes.

Mir-15a has been implicated in a variety of pathological events. Specifically, miR-15a is repressed during the development of liver fibrosis and liver cancer 29, 30. Another important finding of this study was the discovery of miR-15a as a regulator located downstream from the Gα_12_ pathway in normal hepatocytes. Moreover, miR-15a levels were decreased in animals subjected to either APAP treatment or Gα_12_ overexpression, whereas the opposite occurred when Gα_12_ was ablated. The outcomes of this study demonstrated that miR-15a directly interacts with the 3'-UTR of ALOX12 mRNA, thereby inhibiting ALOX12. The negative relationship between miR-15a and Gα_12_ was corroborated using human liver samples, providing a new clinical basis for the targeting of specific disease-associated molecules.

Lipid peroxides are also removed by GPX4 and its cofactor GSH. GPX4 inactivation causes GSH depletion through lipid hydroperoxide accumulation 31, highlighting the key role of this enzyme in regulating cell death control. Moreover, APAP-induced Gα_12_ overexpression affects several gene clusters such as superoxide generation, arachidonic acid binding, and biosynthesis of unsaturated fatty acids, all of which may favor the supply of PUFAs. In line with these observations, ferroptosis inducers led to Gα_12_ overexpression. The cyclic link between Gα_12_ overexpression and GPX4 inhibition was further strengthened by the fact that APAP-triggered lipid peroxidation was alleviated in the liver of *Gna12* KO mice or by Gα_12_ silencing. Together, our findings strongly support the hypothesis that Gα_12_ overexpression by ER stress facilitates lipid peroxidation through GPX4 inhibition. It has been shown that interaction between ALOX12 and SLC7A11 was unaffected by Erastin or other ferroptosis inducers, or by GPX4 inhibition 32. Thus, GPX4 and ALOX12 may independently mediate ferroptosis.

APAP poisoning and fatalities are linked to increased production of reactive metabolites 33. Currently,* N*-acetylcysteine is used to treat APAP-intoxicated patients. However, its efficacy is insufficient to treat patients with acute liver injury, and this antidote has other limitations 34. Therefore, new therapeutic agents must be identified. The role of ROCK inhibitors in a variety of diseases has been widely investigated 35. Here, inhibitors of ROCK1, but not ROCK2, effectively ameliorated acute liver injury. Moreover, ROCK1 inhibition increased GPX4 levels while also inhibiting ALOX12. Therefore, it is likely that ROCK1-dependent changes in GPX4 and ALOX12 levels contribute to APAP toxicity. Our findings highlight the potential applications of ROCK1 inhibitors for the development of novel approaches to treat acute liver injury and related liver diseases.

Repetitive or long-term exposure to toxicants may lead to the development of hepatitis and liver fibrosis 36. Previously, we showed that Gα_12_ is overly expressed in activated hepatic stellate cells, thus promoting liver fibrosis through autophagy 37. In the present human fibrotic sample analyses, Gα_12_ expression correlated well with that of ALOX12, as well as reciprocal changes in GPX4 and miR-15a levels. This outcome was further supported by additional data showing that *ROCK1* transcripts were markedly upregulated in patients with acute liver failure or cirrhosis, suggesting that the Gα_12_-ROCK1 axis may culminate in ferroptosis and related liver diseases in clinical settings. Additionally, the correlations existed between *Gna12* and *Ddit3* (or *Alox12/Gpx4*) levels in the datasets obtained from hepatocytes or liver tissues exposed to tolvaptan, diclofenac, and methapyrilene (Figure S1C and S8).

In the present study, we analyzed the dataset originated from hepatocytes to obtain information on hepatocyte biology excluding the influences of non-parenchymal cell mediators 38. Liver tissue was also used for RNA sequencing in addition to GEO dataset analyses, which is necessary to understand *in vivo* physiology. HepG2 cell line was employed for signaling pathway mapping. All of these models consistently show comparable outcomes, supporting our hypothesis. Collectively, our findings demonstrate that overexpression of Gα_12_ in hepatocytes by ER stress induces ALOX12 through ROCK1 by dysregulating miR-15a. This, in turn, promotes hepatocyte ferroptosis during the event of acute liver injury, and also presumably leads to the progression of fibrosis. Therefore, pharmacological inhibition of the Gα_12_-ROCK1 axis may represent a promising strategy for the treatment of liver diseases including acute liver injury, hepatitis, and fibrosis/cirrhosis.

## Supplementary Material

Supplementary materials and methods, figures and tables.

## Abbreviations

4-HNE: 4-hydroxynonenal
3-NT: 3-nitrotyrosine
Ad: adenovirus
ALOX12: arachidonate 12-lipoxygenase
ALT: alanine aminotransferase
ANOVA: analysis of variance
APAP: acetaminophen
ASO: antisense oligonucleotide
AST: aspartate aminotransferase
ATF6: activating transcription factor 6
BSO: buthionine sulfoximine
CASP1: caspase 1
CCl_4_: carbon tetrachloride
DILI: drug induced liver injury
ER: endoplasmic reticulum
FDR: false discovery rate
Fer-1: ferrostatin-1
Gα_12_: G protein subunit alpha 12
GEO: Gene Expression Omnibus
GFP: green fluorescence protein
GO: Gene Ontology
GPCR: G protein-coupled receptors
GPX4: glutathione peroxidase 4
GRP78: glucose regulatory protein 78
GSH: glutathione
GSEA: gene set enrichment analysis
IRE1α: inositol-requiring enzyme 1α
KEGG: Kyoto Encyclopedia of Genes and Genomes
KO: knockout
MHN: massive hepatic necrosis
miR-15a: microRNA-15a
miRNA: microRNA
MLKL: mixed lineage kinase domain like pseudokinase
NAC: N-acetylcysteine
NAPQI: N-acetyl-p-benzoquinone imine
NES: normalized enrichment score
PERK: protein kinase R-like endoplasmic reticulum kinase
PI: propidium iodide
PUFAs: polyunsaturated fatty acids
RhoA: ras homolog gene family member a
RIPK3: receptor-interacting serine/threonine-protein kinase 3
RNA-seq: RNA sequencing
ROCK1: rho associated coiled-coil containing protein kinase 1
ROCK2: rho associated coiled-coil containing protein kinase 2
ROS: reactive oxygen species
SHN: submassive hepatic necrosis
siRNA: small interfering RNA
Tm: tunicamycin
TUNEL: terminal deoxynucleotidyl transferase dUTP nick end labeling
UTR: untranslated region
WT: wild type
Xbp1: the X-box binding protein 1

## References

[1] Bernal W, Wendon J (2013). Acute liver failure. N Engl J Med.

[2] Andrade RJ, Chalasani N, Björnsson ES, Suzuki A, Kullak-Ublick GA, Watkins PB (2019). Drug-induced liver injury. Nature Reviews Disease Primers.

[3] Kang KW, Choi SY, Cho MK, Lee CH, Kim SG (2003). Thrombin induces nitric-oxide synthase via Galpha12/13-coupled protein kinase C-dependent I-kappaBalpha phosphorylation and JNK-mediated I-kappaBalpha degradation. J Biol Chem.

[4] Kim TH, Yang YM, Han CY, Koo JH, Oh H, Kim SS (2018). Gα12 ablation exacerbates liver steatosis and obesity by suppressing USP22/SIRT1-regulated mitochondrial respiration. J Clin Invest.

[5] Dara L, Ji C, Kaplowitz N (2011). The contribution of endoplasmic reticulum stress to liver diseases. Hepatology.

[6] Malhi H, Kaufman RJ (2011). Endoplasmic reticulum stress in liver disease. J Hepatol.

[7] Koo JH, Lee HJ, Kim W, Kim SG (2016). Endoplasmic Reticulum Stress in Hepatic Stellate Cells Promotes Liver Fibrosis via PERK-Mediated Degradation of HNRNPA1 and Up-regulation of SMAD2. Gastroenterology.

[8] Kim JY, Garcia-Carbonell R, Yamachika S, Zhao P, Dhar D, Loomba R (2018). ER Stress Drives Lipogenesis and Steatohepatitis via Caspase-2 Activation of S1P. Cell.

[9] Dixon SJ, Lemberg KM, Lamprecht MR, Skouta R, Zaitsev EM, Gleason CE (2012). Ferroptosis: an iron-dependent form of nonapoptotic cell death. Cell.

[10] Kagan VE, Mao G, Qu F, Angeli JP, Doll S, Croix CS (2017). Oxidized arachidonic and adrenic PEs navigate cells to ferroptosis. Nat Chem Biol.

[11] Wang H, An P, Xie E, Wu Q, Fang X, Gao H (2017). Characterization of ferroptosis in murine models of hemochromatosis. Hepatology.

[12] Friedmann Angeli JP, Schneider M, Proneth B, Tyurina YY, Tyurin VA, Hammond VJ (2014). Inactivation of the ferroptosis regulator Gpx4 triggers acute renal failure in mice. Nat Cell Biol.

[13] Gu JL, Müller S, Mancino V, Offermanns S, Simon MI (2002). Interaction of G alpha(12) with G alpha(13) and G alpha(q) signaling pathways. Proc Natl Acad Sci U S A.

[14] Mohar I, Stamper BD, Rademacher PM, White CC, Nelson SD, Kavanagh TJ (2014). Acetaminophen-induced liver damage in mice is associated with gender-specific adduction of peroxiredoxin-6. Redox Biol.

[15] Han CY, Lim SW, Koo JH, Kim W, Kim SG (2016). PHLDA3 overexpression in hepatocytes by endoplasmic reticulum stress via IRE1-Xbp1s pathway expedites liver injury. Gut.

[16] Xie Y, Hou W, Song X, Yu Y, Huang J, Sun X (2016). Ferroptosis: process and function. Cell Death Differ.

[17] Stockwell BR, Friedmann Angeli JP, Bayir H, Bush AI, Conrad M, Dixon SJ (2017). Ferroptosis: A Regulated Cell Death Nexus Linking Metabolism, Redox Biology, and Disease. Cell.

[18] Su L-J, Zhang J-H, Gomez H, Murugan R, Hong X, Xu D (2019). Reactive Oxygen Species-Induced Lipid Peroxidation in Apoptosis, Autophagy, and Ferroptosis. Oxid Med Cell Longev.

[19] Wang K, Zhang S, Marzolf B, Troisch P, Brightman A, Hu Z (2009). Circulating microRNAs, potential biomarkers for drug-induced liver injury. Proc Natl Acad Sci U S A.

[20] Riobo NA, Manning DR (2005). Receptors coupled to heterotrimeric G proteins of the G12 family. Trends Pharmacol Sci.

[21] Woolbright BL, Jaeschke H (2017). Role of the inflammasome in acetaminophen-induced liver injury and acute liver failure. J Hepatol.

[22] Ramachandran A, Jaeschke H (2019). Acetaminophen Hepatotoxicity. Semin Liver Dis.

[23] Jaeschke H, Duan L, Akakpo JY, Farhood A, Ramachandran A (2018). The role of apoptosis in acetaminophen hepatotoxicity. Food Chem Toxicol.

[24] Dara L, Johnson H, Suda J, Win S, Gaarde W, Han D (2015). Receptor interacting protein kinase 1 mediates murine acetaminophen toxicity independent of the necrosome and not through necroptosis. Hepatology.

[25] Yamada N, Karasawa T, Kimura H, Watanabe S, Komada T, Kamata R (2020). Ferroptosis driven by radical oxidation of n-6 polyunsaturated fatty acids mediates acetaminophen-induced acute liver failure. Cell Death Dis.

[26] Yang WS, Kim KJ, Gaschler MM, Patel M, Shchepinov MS, Stockwell BR (2016). Peroxidation of polyunsaturated fatty acids by lipoxygenases drives ferroptosis. Proc Natl Acad Sci U S A.

[27] Zhang XJ, Cheng X, Yan ZZ, Fang J, Wang X, Wang W (2018). An ALOX12-12-HETE-GPR31 signaling axis is a key mediator of hepatic ischemia-reperfusion injury. Nat Med.

[28] Chu B, Kon N, Chen D, Li T, Liu T, Jiang L (2019). ALOX12 is required for p53-mediated tumour suppression through a distinct ferroptosis pathway. Nature Cell Biology.

[29] Jiang XP, Ai WB, Wan LY, Zhang YQ, Wu JF (2017). The roles of microRNA families in hepatic fibrosis. Cell Biosci.

[30] Xin X, Wu M, Meng Q, Wang C, Lu Y, Yang Y (2018). Long noncoding RNA HULC accelerates liver cancer by inhibiting PTEN via autophagy cooperation to miR15a. Mol Cancer.

[31] Yang WS, SriRamaratnam R, Welsch ME, Shimada K, Skouta R, Viswanathan VS (2014). Regulation of ferroptotic cancer cell death by GPX4. Cell.

[32] Pan X, Lin Z, Jiang D, Yu Y, Yang D, Zhou H (2019). Erastin decreases radioresistance of NSCLC cells partially by inducing GPX4-mediated ferroptosis. Oncol Lett.

[33] Kaplowitz N (2005). Idiosyncratic drug hepatotoxicity. Nat Rev Drug Discov.

[34] Smilkstein MJ, Knapp GL, Kulig KW, Rumack BH (1988). Efficacy of oral N-acetylcysteine in the treatment of acetaminophen overdose. Analysis of the national multicenter study (1976 to 1985). N Engl J Med.

[35] Hahmann C, Schroeter T (2010). Rho-kinase inhibitors as therapeutics: from pan inhibition to isoform selectivity. Cell Mol Life Sci.

[36] Bai Q, Yan H, Sheng Y, Jin Y, Shi L, Ji L (2017). Long-term acetaminophen treatment induced liver fibrosis in mice and the involvement of Egr-1. Toxicology.

[37] Kim KM, Han CY, Kim JY, Cho SS, Kim YS, Koo JH (2018). Gα(12) overexpression induced by miR-16 dysregulation contributes to liver fibrosis by promoting autophagy in hepatic stellate cells. J Hepatol.

[38] Racanelli V, Rehermann B (2006). The liver as an immunological organ. Hepatology.

