# Supplementary material for: Gα12 overexpression in hepatocytes by ER stress exacerbates acute liver injury via ROCK1-mediated miR-15a and ALOX12 dysregulation
Source: Theranostics. 2022 Jan 9;12(4):1570–88. doi: 10.7150/thno.67722 (PMC8825599; doi:10.7150/thno.67722)
Supplement: Supplementary file 1 — Supplementary materials and methods, figures and tables. [file thnov12p1570s1.pdf]

SUPPLEMENTAL MATERIALS

$G\alpha_{12}$ overexpression in hepatocytes by ER stress exacerbates acute liver injury via ROCK1-mediated miR-15a and ALOX12 dysregulation

Jihoon Tak[1], Yun Seok Kim[2], Tae Hyun Kim[1,3,4], Gil-Chun Park[5], Shin Hwang[5], and Sang Geon Kim[6,*]

Table of contents

Materials and Methods

Supplementary References

Fig. S1. Correlation between *Gna12* and ER stress marker expression in liver injury models

Fig. S2. Effects of $G\alpha_{12}$ knockdown on 4-HNE adducts formation

Fig. S3. An inverse correlation between $G\alpha_{12}$ and GPX4 levels upon ferroptosis inducers

Fig. S4. Effects of Fer-1 on hepatocyte injury and $G\alpha_{12}$ expression

Fig. S5. Effects of $G\alpha_{12}$ modulation on *Alox12* and GPX4 levels in the liver of mice treated with APAP

Fig. S6. Effects of ROCK1/2 inhibition on ALOX12 in cells treated with APAP

Fig. S7. Effects of $G\alpha_{12}$ modulations on liver inflammation and hepatocyte death

Fig. S8. Analysis of hepatic *Gna12*, *Alox12*, and *Gpx4* transcripts in rats treated with methapyrilene using GEO database

Supplementary Table 1

Supplementary Table 2

Supplementary Materials and Methods

Materials

Antibodies directed against $G\alpha_{12}$ (sc-409, sc-515445), IRE1α (sc-390960), GPX4 (sc-166570), and ALOX12 (sc-365194) were purchased from Santa Cruz Biotechnology (Santa Cruz, CA). Anti-GRP78 (ab21685), anti-4-hydroxynonenal (ab46545), anti-3-nitrotyrosine (ab61392), and anti-ROCK1 (ab45171) antibodies were supplied from Abcam (Cambridge, UK), whereas anti-phospho PERK (Thr980) (3179), anti-ATF6 (65880), anti-XBP1s (83418S), anti-phospho MLC (91689), anti-ROCK2 (9029) antibodies were from Cell Signaling Technology (Danvers, MA). BODIPY 581/591 C11, horseradish peroxidase-conjugated goat anti-rabbit (G-21234), and goat anti-mouse (G-21040) IgGs were purchased from Invitrogen (Carlsbad, CA). Y-27632 (1254), ripasudil (K-115) hydrochloride dehydrate (S7995), and netarsudil (AR-13324) (B7807) were obtained from Tocris Biosciences (Bristol, UK), Selleckchem (Houston, TX), and APExBIO (Houston, TX), respectively. Anti-β-actin antibody (A5441) and other reagents were purchased from Sigma-Aldrich (St. Louis, MO).

Analysis of human samples

For the analysis of drug-induced liver injury (DILI) samples, human liver samples were obtained from donors and recipients undergoing liver transplantation from 2011 to 2020 after histologic examination and ultrasonography at Asan Medical Center (Seoul, South Korea) (IRB no. 2021-0839). During the donor sample procurement, an intraoperative assessment of the liver was systematically carried out to rule out fibrosis, cirrhosis, steatosis, and other abnormalities before transplantation (Supplementary Table 1). Human liver samples of normal individuals (n = 5) or DILI patients (n = 22) were processed for RNA isolation, immunoblottings for $G\alpha_{12}$, ALOX12, and qRT-PCR assays for miR-15a. All patients in this study provided written informed consent, and the study was approved by the institutional review board of Asan Medical Center.

For the human fibrosis analysis, nontumorous liver tissues adjacent to liver cancer were collected from patients who had been diagnosed with liver fibrosis or cirrhosis by histologic examination between

2006 and 2009 in Asan Medical Center (Seoul, Korea) (IRB no. 2012-0133) [1]. After resection, fresh surgical specimens were immediately snap-frozen in liquid nitrogen and stored at −80 ºC. Informed consent from the patients was obtained before operations and the study protocol was approved by the institutional review board of Asan Medical Center in accordance with the ethical guidelines of the 1975 Declaration of Helsinki. Ethics approval was provided by the ethics committees of Seoul National University. G protein subunit alpha 12 ($G\alpha_{12}$), arachidonate 12-lipoxygenase (ALOX12), glutathione peroxidase 4 (GPX4), and microRNA-15a (miR-15a) levels were measured among normal (n = 2), portal fibrosis (n = 10), septal fibrosis (n = 15), and cirrhosis (n = 20) groups.

RNA seq analysis

For RNA-seq analysis, liver from either WT or *Gna12* KO mice treated with APAP (300 mg/kg BW, i.p.) or vehicle were used (n = 3 each). The sampling protocol was applied in *Gna12* KO mice and their age-matched WT littermates. Total RNA concentration was calculated by Quant-IT RiboGreen (Thermo Fisher Scientific, R11490). To assess the integrity of the RNA, samples were run on the Tape Station RNA screen tape (Agilent Technologies, 5067-5576). Only high-quality RNA preparations, with RIN N7.0, were used for RNA library construction. A library was independently prepared with 1 μg total RNA for each sample by Illumina TruSeq Stranded mRNA Sample Prep Kit (Illumina, Inc., RS-122-2101). The first step in the workflow involved purifying the poly-A containing mRNA molecules using poly-T-attached magnetic beads. Following purification, the mRNA was fragmented into small pieces using divalent cations under elevated temperature. The cleaved RNA fragments were copied into first-strand cDNA using SuperScript II reverse transcriptase (Invitrogen, 18064014) and random primers. This was followed by second-strand cDNA synthesis using DNA polymerase I, RNase H, and dUTP. These cDNA fragments then went through an end repair process, the addition of a single 'A' base, and then ligation of the adapters. The products were purified and enriched with PCR to create the final cDNA library. The libraries were quantified using KAPA Library Quantification Kits for Illumina Sequencing platforms according to the qPCR Quantification Protocol Guide (Kapa Biosystems,

KK4854) and qualified using the TapeStation D1000 ScreenTape (Agilent Technologies, 5067-5582). Indexed libraries were submitted to an Illumina NovaSeq 6000 (Illumina, Inc.), and the paired-end (2×100 bp) sequencing was done by Macrogen Incorporated. The raw transcriptomics data have been deposited in the Gene Expression Omnibus (GEO) under the accession code GSE173595.

Bioinformatic analysis

For RNA-seq analysis, the raw 'Transcripts Per Kilobase Million (TPM)' values were processed and normalized by logarithm and quantile normalization method. RNA-seq data from WT mice and *Gna12* KO mice treated with APAP were analyzed using Gene Set Enrichment Analysis (GSEA) 3.0 software. 'Biological process of gene ontology (GO)' from Molecular Signature Database (MSigDB, http://software.broadinstitute.org/gsea/msigdb) v6.2 was employed. False discovery rate (FDR) was used for statistical significance assessment of normalized enrichment score (NES). Gene sets with FDR values < 0.05 or 0.25 were considered statistically significant. Heat map represents the respective leading-edge subsets of the most upregulated genes. Publicly accessible patients or mouse gene expression data were downloaded from GEO database (https://www.ncbi.nlm.nih.gov/geo/; GSE2082, GSE51694, GSE74000, GSE44079, GSE38941, GSE25097, GSE40336, GSE104302, GSE99878, GSE75277, GSE29929, and GSE95470). Differentially expressed genes (DEGs) were then identified using an independent *t*-test: DEGs were selected as the genes with *P*-values < 0.001 or 0.05 with a fold-change of ≥ 1.5, 4, or ≤ 1.5. Statistically enriched signaling pathways of clustered DEGs were ranked and categorized according to the KEGG pathway using DAVID 6.8 software (https://david.ncifcrf.gov/). Each gene represented by an individual dot in a volcano plot and a heat map of significantly expressed genes were obtained by R software (version 3.6.0) using ggplot and gplots function for the visualization

Target gene delivery

Adenovirus encoding mouse $G\alpha_{12}$QL (Q229L) was kindly provided from Dr. Patrick J. Casey (Duke

University Medical Center, Durham, NC). The mouse albumin enhancer/promoter construct, kindly provided by Dr. Richard D. Palmiter (University of Washington), was used to make a lentivirus encoding $G\alpha_{12}$; the original elongation factor-1 promoter of pCDH-EF1-multiple cloning site-copepod super green fluorescent protein (copGFP) plasmid (System Biosciences) was replaced with the albumin enhancer/promoter. The coding region of pcDNA3-$G\alpha_{12}$ was extracted and was cloned downstream of the albumin enhancer/promoter, as described previously [2]. The constructs were sequenced to assess the integrity of the insert. For *in vivo* experiments, 100 μl of 1.5×10^7 TU was administered to 10-week-old WT or *Gna12* KO mice through the tail vein. At 7 days after injection, the mice were fasted overnight prior to a single dose of APAP treatment, and tissue samples were acquired 6 h later.

Cell lines and primary hepatocytes

AML12 cells (a mouse hepatocyte-derived cell line) were purchased from American Type Culture Collection (ATCC) (Rockville, Maryland), and were cultured in the DMEM/F-12 medium containing 10% FBS, insulin-transferrin-selenium X (ITSX), dexamethasone (40 ng/ml; Sigma), and the antibiotics. The cells with less than 20 passage numbers were used. Primary hepatocytes were isolated from C57BL/6 mice under the guidelines of the institutional animal use and care committee, as described previously [3], and plated in a 6-well dish at a density of 2×10^5 cells/well, and wells with 70% to 80% confluence were used. Briefly, under anesthesia with Zoletil, livers were perfused with Ca^{2+}-free Hank's buffered salt solution (Invitrogen, Carlsbad, CA) for 10 min, followed by continuous perfusion with a 0.1% w/v collagenase (Sigma, Type I). The whole liver was removed, and minced in the phosphate-buffered saline. Mouse hepatocytes were filtered through a 0.2 μm cell strainer (BD Biosciences) and centrifuged at 50 *g* for 2 min (three times) to separate hepatocytes. Hepatocytes were harvested into collagen-coated plates in isolation media (Dulbecco's modified Eagle's medium [DMEM], high glucose, supplemented with 10% fetal bovine serum [FBS], 100 units/mL penicillin and 100 μg/mL streptomycin, 15 mM 4-(2-hydroxyethyl) piperazine-1-ethanesulfonic acid [HEPES], and 10 nM dexamethasone). After 3 to 4 h, unattached cells were removed by changing isolation media with

culture media (DMEM, low glucose, supplemented with 10% FBS, 100 units/mL penicillin, and 100 μg/mL streptomycin, 5 mM HEPES, and 10 nM dexamethasone). The attached hepatocytes were used for further analysis.

RNA isolation and quantitative RT-PCR assays

Total RNA was extracted using Trizol (Invitrogen, Carlsbad, CA) and was reverse-transcribed. The resulting cDNA was amplified by qRT-PCR as previously described [1]. β-actin or 18S rRNA or GAPDH was used as the normalization control. qRT-PCR assays for miRNA, cDNA was generated from equal amounts of total RNA per sample (1 μg) using the miScript Reverse Transcription kit (Qiagen GmbH, Hilden, Germany). The reaction mixture containing reverse transcription product, 2x QuantiTect SYBR Green PCR Master Mix, 10x miScript Universal Primer, and primers were incubated at 95ºC for 15 min, followed by 40 amplification cycles of 94ºC for 10 s, 55ºC for 30 s, and 70ºC for 30 s. The threshold cycle (Ct) was defined as the fractional cycle number at which the fluorescence passed the fixed threshold. Transcripts of U6 small RNA were quantified using the Hs_RNU6B_2 miScript Primer Assay (Qiagen, Hilden, Germany) for normalization of miRNA levels. The relative expression values were normalized to the internal control using $2^{-\Delta\Delta Ct}$. The primer sequences used for qRT-PCR assays were listed in Supplementary Table 2.

Plasmid and small interfering RNA transfection

The plasmids encoding for XBP1s and IRE1α were supplied from Addgene (Cambridge, MA). The empty plasmid, pcDNA3.1, was used for mock transfection. Cells were seeded in 6-well plates and were incubated with the indicated plasmid (1 μg) for transfection using Lipofectamine 2000 (Invitrogen, Carlsbad, CA) according to the manufacturer's instructions. Scrambled control siRNA or siRNA directed against $G\alpha_{12}$, siIRE1α, siPERK, siATF6, siXBP1, siROCK1, and siROCK2 were supplied from Dharmacon (Lafayette, CO, USA). The cells were transfected with each small-interfering RNA (siRNA) (100 nM) using Lipofectamine 2000 transfection reagent (Invitrogen, Carlsbad, CA) for 24 h.

Luciferase reporter assays

For reporter gene assays, the region containing -2,000 base pair (bp) of *Gna12* was cloned into the pGL3 luciferase vector (pGL3-Gna12). A mutation of Xbp1-RE was done by replacing the sequence of Xbp1 binding element from 5'-CATCTCTAG-3' to 5'-CAT**AAAA**AG-3' (bolds indicate mutation). Cells were transfected with either pGL3-Gα_{12} reporter or pGL3-Gα_{12}-Xbp1-RE mutant reporter for 24 h in the presence of Lipofectamine 2000 transfection reagent (Invitrogen, Carlsbad, CA), and luciferase activity was measured by adding luciferase assay reagent (Promega, Madison, WI).

Adenoviral infection

Adenovirus encoding for mouse Gα_{12}QL (Q229L) was kindly provided by Dr. Patrick J. Casey (Duke University Medical Center, Durham, NC). For *in vitro* assays using adenovirus, Ad-GFP was used as infection control.

Immunoblot analysis

Cells were centrifuged at 3,000 *g* for 3 min and allowed to swell after the addition of lysis buffer in ice for 1 h. The lysates were centrifuged at 10,000 *g* for 10 min to obtain supernatants. Proteins were separated by 6%, 7.5%, or 12% sodium dodecyl sulfate-polyacrylamide gel electrophoresis and were transferred onto nitrocellulose membranes (Millipore, Bedford, MA). The membrane was blocked with 5% non-fat dried milk in Tris-buffered saline and Tween 20 (TBST) (20 mM Tris-HCl, 150 mM NaCl, and 0.1% Tween 20, pH 7.5) for 1 h, and incubated overnight with primary antibodies at 4ºC. After washing with TBST buffer, membranes were incubated with a horseradish peroxidase-conjugated anti-mouse IgG secondary antibody for 1 h at room temperature. Bands were visualized using the ECL chemiluminescence system (GE Healthcare, Buckinghamshire, UK). Equal loading of proteins was verified by immunoblotting for β-actin. Quantifications were done by scanning densitometry of the immunoblots and β-actin normalization.

Immunohistochemistry

Formalin-fixed, paraffin-embedded human fibrotic liver samples were cut using a microtome into 4-μm thickness. The tissue sections were mounted on glass slides. Antibodies of interest ($G\alpha_{12}$, ALOX12, and GPX4) were incubated overnight at room temperature with the slide-mounted method. Mouse liver specimens were fixed in 10% formalin, embedded in paraffin, cut into sections, and were mounted on slides. Tissue sections were immunostained with the antibodies directed against $G\alpha_{12}$, GRP78, 4-HNE, ALOX12, and GPX4. Briefly, the paraffin-embedded sections were deparaffinized with xylene and rehydrated with alcohol series. After antigen retrieval was performed, the endogenous peroxidase activity was quenched. The sections were pretreated with 10% normal donkey serum for 40 min to block nonspecific antibody binding and incubated with the antibodies of interest overnight at 4 °C. The sections were then treated with 2% normal donkey serum for 15 min and incubated with biotin-SP-conjugated affinity pure donkey anti-rabbit IgG for 2 h. The labeling was done by using 3,3'-diaminobenzidine. After mounting with Permount solution, the sections were examined using a light microscope (DMRE, Leica Microsystems, Wetzlar, Germany), and images were acquired with Fluoview-II (Soft Imaging System GmbH, Muenster, Germany) attached on the microscope.

Transfection of miRNA mimic or antisense oligonucleotide

Synthetic miRNA duplexes were synthesized and transfected, as previously described [4]. Briefly, the cells in each well (6-well plates) were transiently transfected with 100 nM of miR-15a mimic or control mimic (GenePharma, China), or 2'-O-methyl miR-15a antisense oligonucleotide (ASO) or respective control ASO using Lipofectamine 2000 transfection reagent (Invitrogen, Carlsbad, CA) for 48 h.

Alox12 3'-UTR reporter assays

The miRNA 3'-untranslated region (UTR) target clone (Luc-*Alox12*-3'UTR), which contains Renila luciferase as internal control fused downstream to firefly luciferase, was purchased from

GeneCopoeia (MmiT090956-MT06; Rockville, MD). Luciferase activity assays were done according to manufacturer protocols. Briefly, AML12 cells were seeded in 6-well plates, and co-transfected with Luc-*Alox12*-3'UTR reporter and miR-15a mimic (or ASO) or its relative control using Lipofectamine 2000 transfection reagent (Invitrogen, Carlsbad, CA). The target site within the *Alox12* 3'UTR reporter was used to generate a mutant reporter construct (5'-U**CGCCGC**G-3', bolds indicate mutations), which was used as a negative control. Firefly and Renilla luciferase activities were measured sequentially using the Luc-Pair miR Luciferase Assay kit (GeneCopoeia, Rockville, MD). The activities were normalized with Renilla luciferase activities and expressed in relative luciferase activity units.

Blood biochemical analysis and histopathology

Serum alanine aminotransferase (ALT) and aspartate aminotransferase (AST) activities were analyzed using Spectrum (Abbott Laboratories, Abbott Park, IL). Hematoxylin and eosin (H&E) staining was done as described [5]. Terminal deoxynucleotidyl transferase-mediated deoxyuridine triphosphate nick-end labeling (TUNEL) assays were carried out using the *in situ* S7100 ApopTAG apoptosis detection kit from Millipore (Temecula, CA) and the DeadEnd Colorimetric TUNEL System from Promega (Madison, WI) according to the manufacturer's instructions. For the counting of TUNEL-positive cells, three fields/slides at ×100 magnification were randomly selected, and the percentage of TUNEL-positive staining was assessed using Image J (National Institutes of Health) software. Data were obtained from two to three samples per condition.

Flow cytometric analysis

Apoptosis was analyzed by the FITC-annexin V plus PI staining method according to the published method [6]. Briefly, cells were harvested by trypsinization. After washing with PBS containing 1% FBS, the cells were stained with 5 μl FITC-annexin V and 1 μl PI/~2×10^5 cells. The fluorescence intensity in the cells was assessed using BD fluorescence-activated cell sorting (FACS) Calibur II flow cytometer and the Cell Quest software (BD Biosciences, San Jose, CA). In each analysis, 10,000 gated events

were recorded.

Fluorescence-activated cell sorting of BODIPY staining

C11-BODIPY581/591 was prepared by dissolving 1 mg of the product in dimethylsulfoxide to a concentration of 1 mM. AML12 cells were transfected with siCon or siGα_{12} for 24 h. The Cells were incubated for 30 min at 37 °C with C11-BODIPY581/591 (1 μM) in a growth medium 24 h after 10 mM APAP treatment. After washing with PBS twice, the cells were harvested by trypsinization. The cells were then placed in 1% FBS after centrifugation for 2.5 min. In each analysis, 10,000 gated events were recorded. The fluorescence intensity in the cells was assessed using the FACS Calibur II flow cytometer and the Cell Quest software (BD Biosciences, San Jose, CA).

Supplementary References

1. Yang YM, Lee WH, Lee CG, An J, Kim ES, Kim SH, et al. Gα12 gep oncogene deregulation of p53-responsive microRNAs promotes epithelial-mesenchymal transition of hepatocellular carcinoma. Oncogene. 2015; 34: 2910-21.
2. Kim TH, Yang YM, Han CY, Koo JH, Oh H, Kim SS, et al. Gα12 ablation exacerbates liver steatosis and obesity by suppressing USP22/SIRT1-regulated mitochondrial respiration. J Clin Invest. 2018; 128: 5587-602.
3. Yang YM, Seo SY, Kim TH, Kim SG. Decrease of microRNA-122 causes hepatic insulin resistance by inducing protein tyrosine phosphatase 1B, which is reversed by licorice flavonoid. Hepatology. 2012; 56: 2209-20.
4. Zhang Q, Pu R, Du Y, Han Y, Su T, Wang H, et al. Non-coding RNAs in hepatitis B or C-associated hepatocellular carcinoma: potential diagnostic and prognostic markers and therapeutic targets. Cancer Lett. 2012; 321: 1-12.
5. Han CY, Lim SW, Koo JH, Kim W, Kim SG. PHLDA3 overexpression in hepatocytes by endoplasmic reticulum stress via IRE1-Xbp1s pathway expedites liver injury. Gut. 2016; 65: 1377-88.
6. Yang YM, Lee CG, Koo JH, Kim TH, Lee JM, An J, et al. Gα12 overexpressed in hepatocellular carcinoma reduces microRNA-122 expression via HNF4α inactivation, which causes c-Met induction. Oncotarget. 2015; 6: 19055-69.

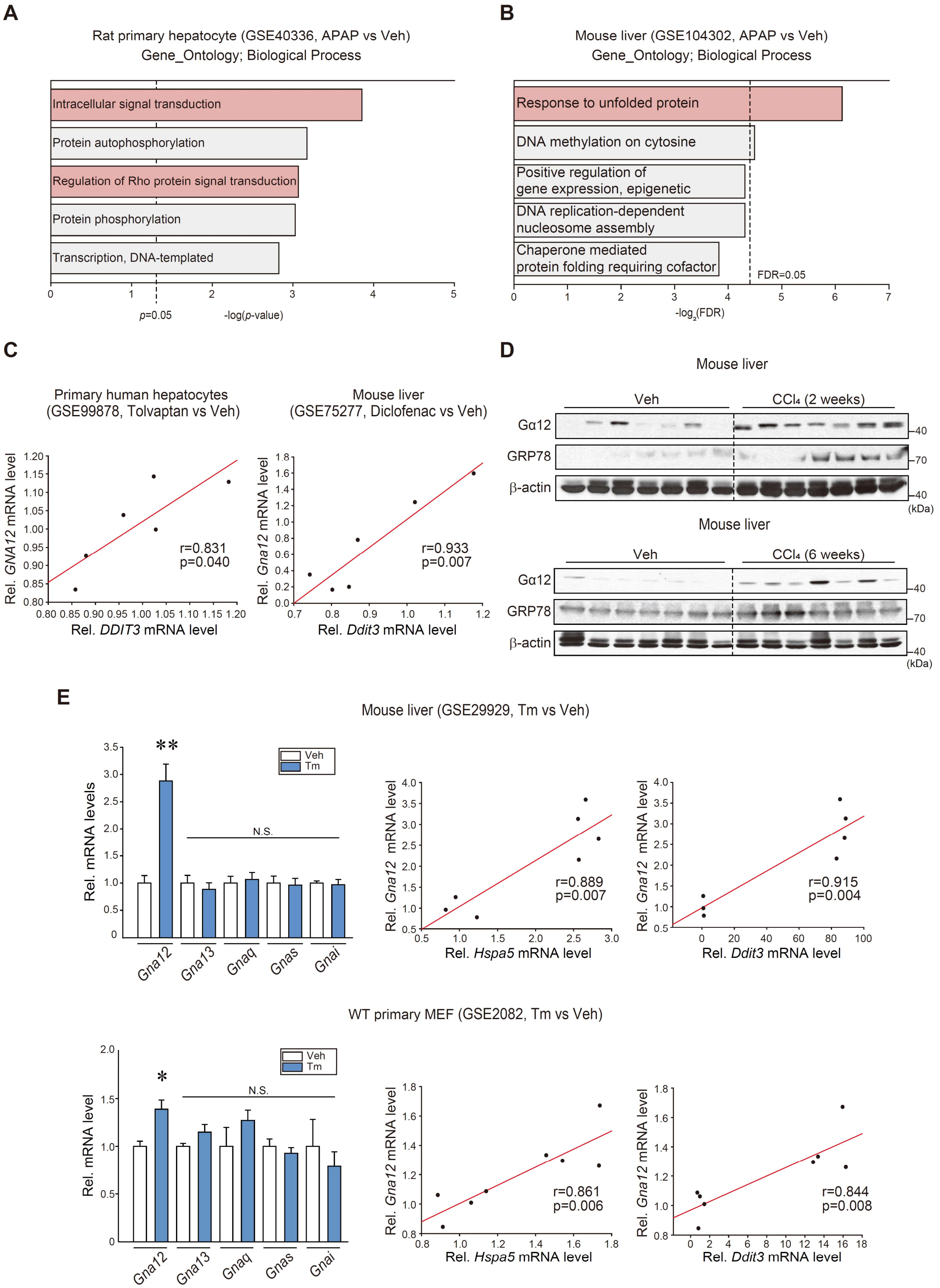
A
Rat primary hepatocyte (GSE40336, APAP vs Veh)
Gene_Ontology; Biological Process
Intracellular signal transduction
Protein autophosphorylation
Regulation of Rho protein signal transduction
Protein phosphorylation
Transcription, DNA-templated
p=0.05
-log(p-value)
B
Mouse liver (GSE104302, APAP vs Veh)
Gene_Ontology; Biological Process
Response to unfolded protein
DNA methylation on cytosine
Positive regulation of gene expression, epigenetic
DNA replication-dependent nucleosome assembly
Chaperone mediated protein folding requiring cofactor
FDR=0.05
-log2(FDR)
C
Primary human hepatocytes (GSE99878, Tolvaptan vs Veh)
Rel. GNA12 mRNA level
Rel. DDIT3 mRNA level
r=0.831
p=0.040
Mouse liver (GSE75277, Diclofenac vs Veh)
Rel. Gna12 mRNA level
Rel. Ddit3 mRNA level
r=0.933
p=0.007
D
Mouse liver
Veh
CCl4 (2 weeks)
Gα12
GRP78
β-actin
(kDa)
Mouse liver
Veh
CCl4 (6 weeks)
Gα12
GRP78
β-actin
(kDa)
E
Mouse liver (GSE29929, Tm vs Veh)
Rel. mRNA levels
Veh
Tm
N.S.
Gna12
Gna13
Gnaq
Gnas
Gnai
Rel. Gna12 mRNA level
Rel. Hspa5 mRNA level
r=0.889
p=0.007
Rel. Gna12 mRNA level
Rel. Ddit3 mRNA level
r=0.915
p=0.004
WT primary MEF (GSE2082, Tm vs Veh)
Rel. mRNA level
Veh
Tm
N.S.
Gna12
Gna13
Gnaq
Gnas
Gnai
Rel. Gna12 mRNA level
Rel. Hspa5 mRNA level
r=0.861
p=0.006
Rel. Gna12 mRNA level
Rel. Ddit3 mRNA level
r=0.844
p=0.008

Supplementary Figure 1. Correlation between *Gna12* and ER stress marker expression in liver injury models

(A) Gene ontology analysis using microarray dataset from rat primary hepatocytes treated with APAP (500 μM, 48 h) (GSE40336). Major top 5 biological processes using the DAVID bioinformatics database. GO terms relevant to cell signaling were presented as pink bar graphs.

(B) Gene ontology analysis of major top 5 biological processes in the liver of mice treated with APAP (300 mg/kg BW, i.p., 6 h). A pink bar graph indicates GO term relevant to ER stress (GSE104302, n = 3 each, FDR < 0.08).

(C) Correlation between *GNA12* and ER stress marker transcript (*DDIT3*) using GSE99878 dataset available in the public domain from primary human hepatocytes treated with tolvaptan (0.1 μM, 4 h) or vehicle (left, n = 3 each) and GSE75277 dataset from the liver of mice treated with diclofenac (30 mg/kg BW, 72 h) or vehicle (right, n = 3 each).

(D) Immunoblottings for $G\alpha_{12}$ in the liver of mice treated with CCl_4 (0.5 mL/kg BW, for 2 or 6 weeks).

(E) Gα subunits transcript levels and the correlation between *Gna12* and ER stress marker transcripts (*Hspa5* and *Ddit3*) using GSE29929 dataset available in the public domain from the livers of mice treated with Tm (1 mg/kg BW, 6 h) or vehicle (upper, n = 3 or 4) and GSE2082 dataset from primary MEF cells treated with Tm (2 μg/ml, 8 h) or vehicle (lower, n = 4 each).

For E, values were expressed as mean ± SD (**P* < 0.05, ***P* < 0.01). Statistical significance was tested via two-tailed Student's *t*-test or Pearson correlations.

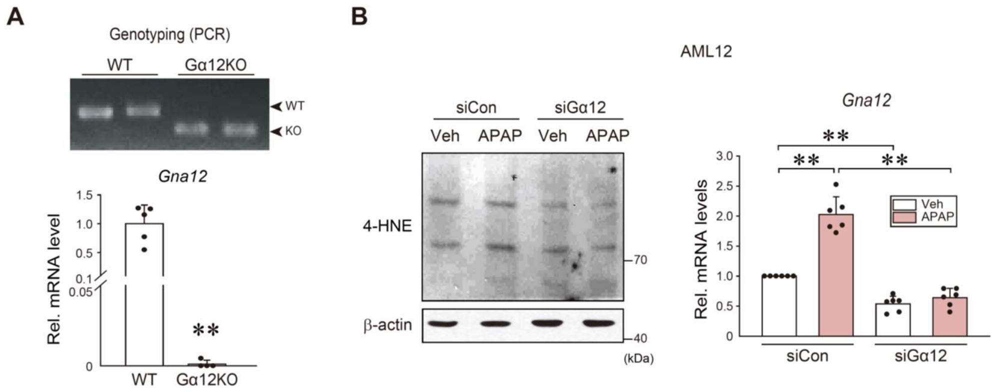

Supplementary Figure 2. Effect of Gα_{12} knockdown on 4-HNE adducts formation

(A) PCR analysis for *Gna12* in genomic DNAs isolated from the tails of WT (littermates) and *Gna12* KO mice (upper). qRT-PCR assay for *Gna12* was done using liver homogenates from the same mice (lower, n = 4 or 5 each).

(B) Immunoblotting for 4-HNE adducts in AML12 cells treated with APAP (10 mM, 12 h) after transfection with siCon or siGα_{12} (100 nM each, 24 h) (left). Validation of siRNA knockdown effect on Gα_{12} (right, n = 6 each).

All values were expressed as mean ± SD (***P* < 0.01). Statistical significance was tested via two-tailed Student's *t*-test or one-way ANOVA coupled with Tukey’s HSD multiple comparison procedure where appropriate.

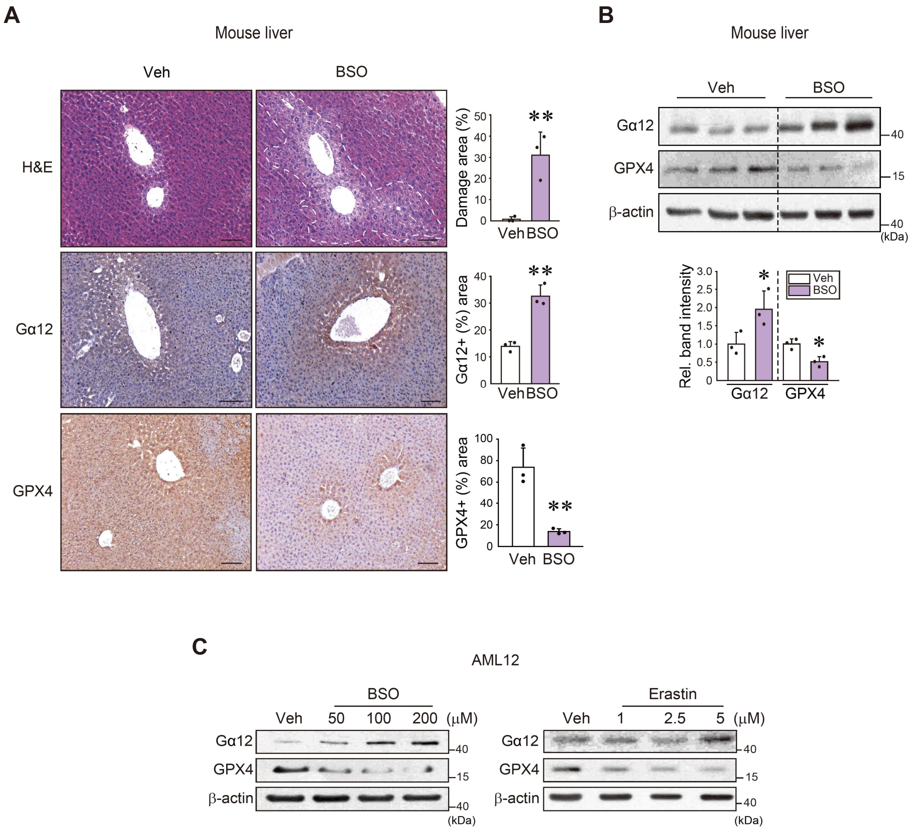

Supplementary Figure 3. An inverse correlation between Gα12 and GPX4 levels upon ferroptosis inducers

(A) Liver histopathology in the livers of mice treated with a single dose of BSO (1 g/kg BW, i.p., 6 h) and immunohistochemistry for $G\alpha_{12}$ and GPX4 (n = 3 each). Percent areas of damaged lesions and $G\alpha_{12}$ staining lesions were measured using the Image J program.

(B) Immunoblottings (upper) for $G\alpha_{12}$ and GPX4 in the livers. The densitometric band intensities represent values relative to their respective controls (lower, n = 3 each).

(C) Immunoblottings for $G\alpha_{12}$ and GPX4 in AML12 cells treated with the indicated concentrations of BSO (n = 2) or Erastin for 12 h (n = 3 each; repeated 3 times with similar results).

For A and B, values were expressed as mean ± SD (**$P < 0.01$). Statistical significance was tested via two-tailed Student's *t*-test.

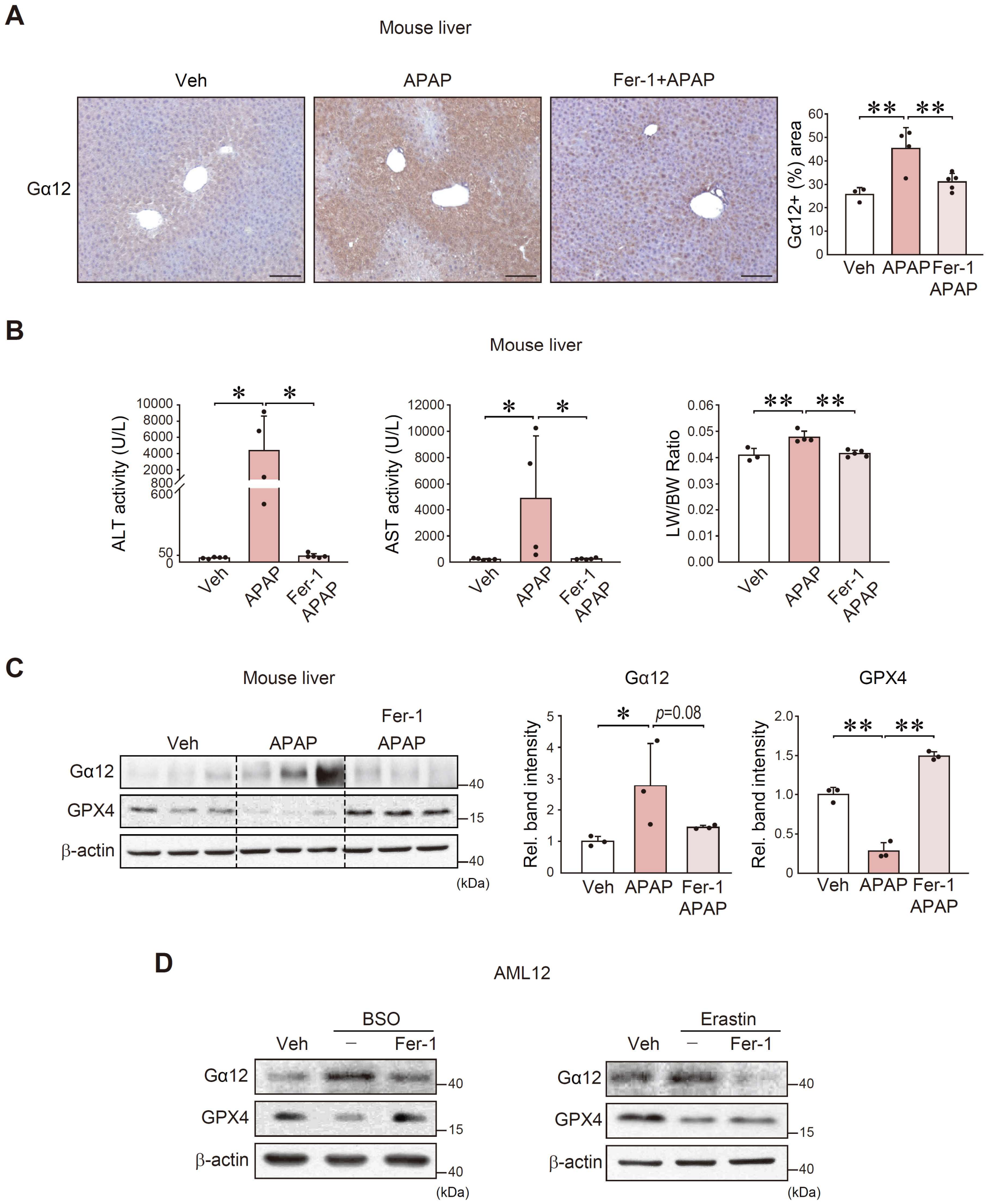

Supplementary Figure 4. Effects of Fer-1 on hepatocyte injury and Gα_{12} expression

(A) Immunohistochemistry for Gα_{12} in the livers of mice treated with APAP (300 mg/kg BW, i.p., 6 h) 1 h after vehicle or Fer-1 (1 mg/kg BW, i.p.) treatment (left). Percent areas of Gα_{12} were assessed using the Image J program (n = 3-5 each) (right). scale bar, 200 μm.

(B) Serum ALT, AST activities, and LW/BW ratios in the same mice as in A (n = 4 or 5 each).

(C) Immunoblottings for $G\alpha_{12}$ and GPX4 in the same mice as in A (left). Densitometric band intensities represent values relative to respective control (right, n = 3 each).

(D) Immunoblottings for $G\alpha_{12}$ and GPX4 in AML12 cells treated with either BSO (200 μM, 12 h) or Erastin (5 μM, 12 h) 1 h after vehicle or Fer-1 treatment (1 μM).

For A-C, values were expressed as mean ± SD (*P < 0.05, **P < 0.01). Statistical significance was tested via one-way ANOVA coupled with Tukey's HSD or the LSD multiple comparison procedures where appropriate.

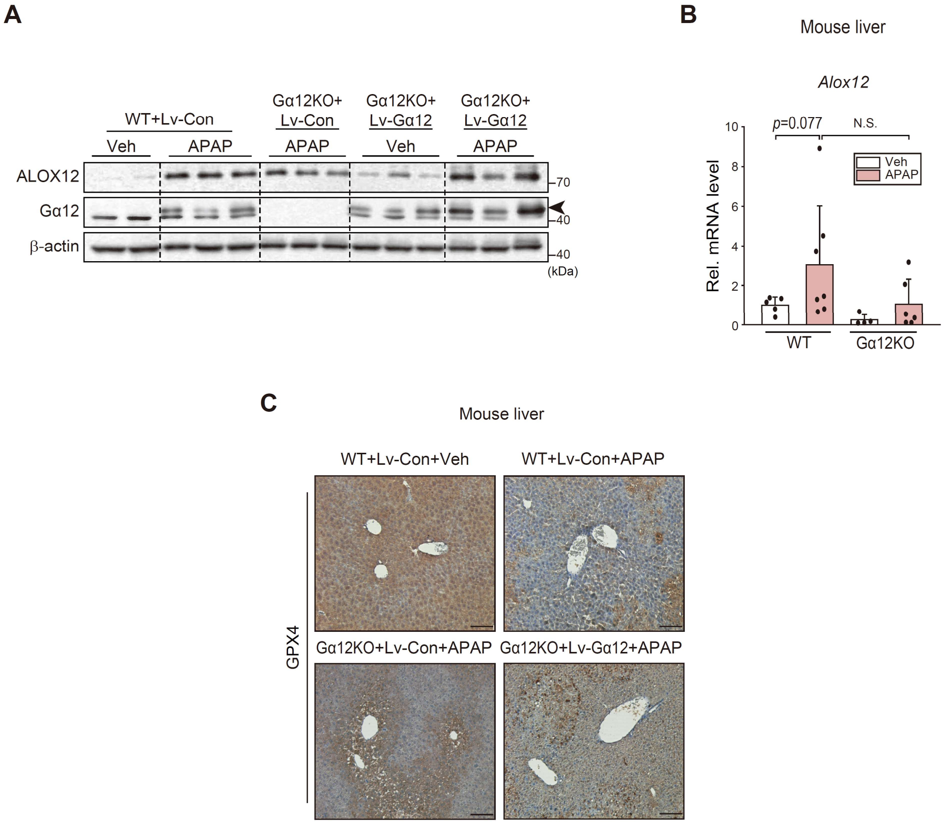

Supplementary Figure 5. Effects of $G\alpha_{12}$ modulation on *Alox12* and GPX4 levels in the liver of mice treated with APAP

(A) Immunoblottings for ALOX12 and $G\alpha_{12}$ in the same mice as in Fig. 4H. *Gna12* KO mice were treated with Veh or APAP 1 week after injection with Lv-Con or Lv-$G\alpha_{12}$ via the tail vein (n = 4-6 each). Arrowhead indicates $G\alpha_{12}$ band.

(B) qRT-PCR assays for *Alox12* in the liver of WT or *Gna12* KO mice treated with vehicle or APAP (300 mg/kg BW, i.p., 6 h) (n = 4-7 each).

(C) Immunohistochemistry for GPX4 in the same samples as in Fig. 4H. Representative liver sections were shown (n = 5-6 each). scale bar, 200 μm.

For B, values were expressed as mean ± SD. Statistical significance was tested via one-way ANOVA coupled with the LSD multiple comparison procedure where appropriate.

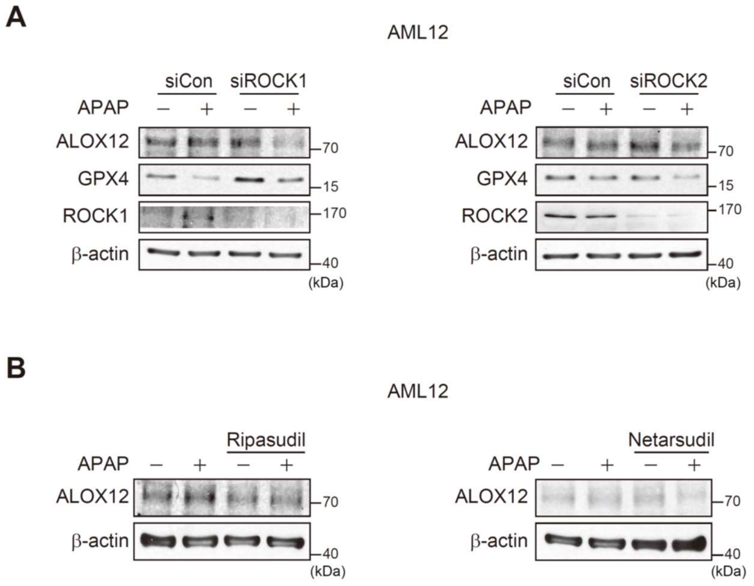

Supplementary Figure 6. Effects of ROCK1/2 inhibition on ALOX12 in cells treated with APAP

(A) Immunoblottings for ALOX12, GPX4, ROCK1, and ROCK2 in AML12 cells treated with APAP (10 mM, 12 h) after transfection with control siRNA (siCon) or siRNA directed against ROCK1 and ROCK2 (100 nM, 24 h).

(B) Immunoblottings for ALOX12 in AML12 cells treated with APAP (10 mM, 12 h) after vehicle, 50 μM ripasudil or 2 μM netarsudil treatment for 1 h.

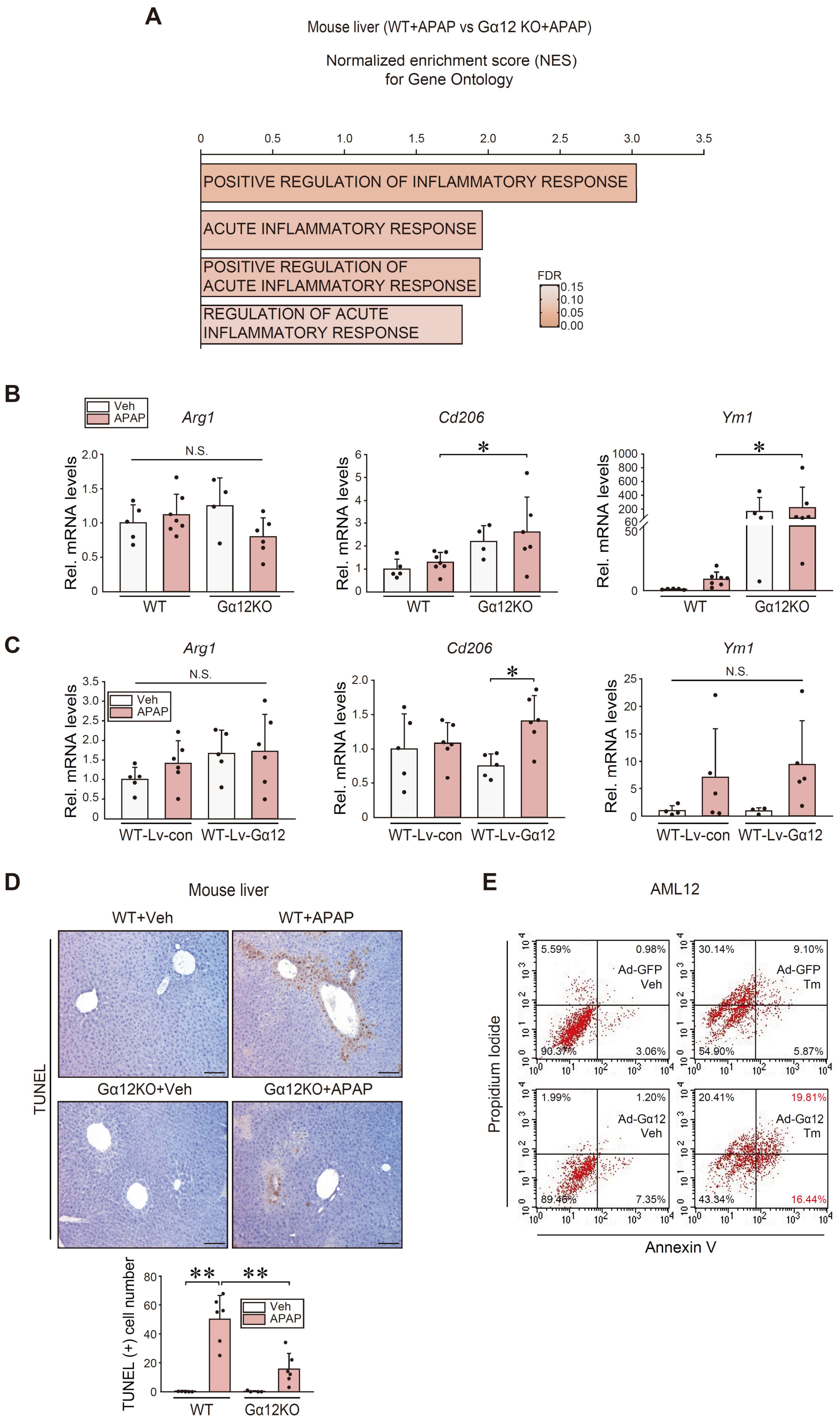
A
Mouse liver (WT+APAP vs Gα12 KO+APAP)
Normalized enrichment score (NES) for Gene Ontology
POSITIVE REGULATION OF INFLAMMATORY RESPONSE
ACUTE INFLAMMATORY RESPONSE
POSITIVE REGULATION OF ACUTE INFLAMMATORY RESPONSE
REGULATION OF ACUTE INFLAMMATORY RESPONSE
FDR
B
Veh
APAP
Arg1
Cd206
Ym1
Rel. mRNA levels
N.S.
WT
Gα12KO
C
WT-Lv-con
WT-Lv-Gα12
D
Mouse liver
WT+Veh
WT+APAP
Gα12KO+Veh
Gα12KO+APAP
TUNEL
TUNEL (+) cell number
E
AML12
Ad-GFP Veh
Ad-GFP Tm
Ad-Gα12 Veh
Ad-Gα12 Tm
Propidium Iodide
Annexin V

Supplementary Figure 7. Effects of Gα_{12} modulations on liver inflammation and hepatocyte death

(A) GO terms associated with inflammatory response positively correlated with WT mice treated with APAP (300 mg/kg BW, i.p., 6 h) compared to the *Gna12* KO mice treated with APAP (n = 3 each). NES and FDR are given as a bar graph.

(B) qRT-PCR assays for inflammatory cytokines (*Arg1, Cd206,* and *Ym1*) in the liver of the same mice as in Fig. 3A (n = 4-7 each).

(C) qRT-PCR assays for inflammatory cytokines (*Arg1, Cd206,* and *Ym1*) in the same mice as in Fig. 4G (n = 3-6 each).

(D) Terminal deoxynucleotidyl transferase dUTP nick end labeling (TUNEL) assays. TUNEL stainings were done on the livers of WT or *Gna12* KO mice treated with a single dose of APAP (300 mg/kg BW, i.p., 6 h) (upper). TUNEL-positive cells were randomly counted from 4-6 samples (lower).

(E) Flow cytometric analyses for fluorescein isothiocyanate-annexin V and propidium iodide in AML12 cells treated with Tm (2 μg/ml, 12 h) 6 h after infection with ad-GFP or ad-Gα_{12}.

For B-D, values were expressed as mean ± SD (*$P < 0.05$, **$P < 0.01$). Statistical significance was tested via one-way ANOVA coupled with Tukey's HSD or the LSD multiple comparison procedures where appropriate.

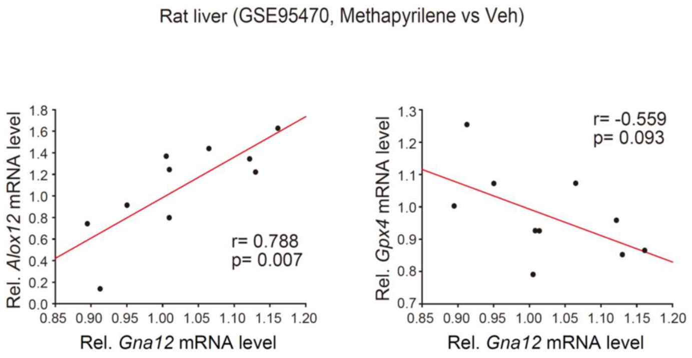

Supplementary Figure 8. Analysis of hepatic *Gna12*, *Alox12*, and *Gpx4* transcripts in rats treated with methapyrilene using GEO database

The positive correlation between *Gna12* and *Alox12* transcripts (left) and the negative correlation between *Gna12* and *Gpx4* (right) using the GSE95470 dataset available in the public domain from the livers of rats treated with methapyrilene (80 mg/kg BW/day, for 8 days, p.o.) or vehicle (n = 5 each).

Data were analyzed by Pearson correlations.

Supplementary Table 1. Characteristics of normal subjects and patients with DILI

<table>
<tr><th colspan="2">Parameters</th><th>Normal
(n = 5)</th><th>DILI
(n = 22)</th><th>P value</th></tr>
<tr><td colspan="2">Age (years)</td><td>27.2 ± 8.2</td><td>46.1 ± 11.7</td><td>0.002</td></tr>
<tr><td colspan="2">Sex (male/female)</td><td>2/3</td><td>9/13</td><td rowspan="7">-</td></tr>
<tr><td colspan="2">Body mass index (kg/m^2)</td><td>25.3 ± 2.8</td><td>24.8 ± 3.1</td></tr>
<tr><td colspan="2">Diabetes (n, %)</td><td rowspan="2">-</td><td>2 (9.1%)</td></tr>
<tr><td colspan="2">Hypertension (n, %)</td><td>5 (22.7%)</td></tr>
<tr><td rowspan="3">Suspected hepatotoxic agents (n, %)</td><td>Herb medication</td><td rowspan="3">-</td><td>7 (31.8%)</td></tr>
<tr><td>Drug medication</td><td>6 (27.3%)</td></tr>
<tr><td>Unknown</td><td>9 (40.9%)</td></tr>
<tr><td rowspan="6">Pretransplant condition</td><td>MELD score</td><td>7.8 ± 1.3</td><td>31.9 ± 7.8</td><td>0.000</td></tr>
<tr><td>CTP score</td><td>5.0</td><td>11.5 ± 1.7</td><td>0.000#</td></tr>
<tr><td>AST (U/L)</td><td>27.4 ± 5.9</td><td>315.6 ± 326.6</td><td>0.000#</td></tr>
<tr><td>ALT (U/L)</td><td>25.4 ± 3.4</td><td>444.3 ± 649.0</td><td>0.003#</td></tr>
<tr><td>Total bilirubin (mg/dL)</td><td>1.02 ± 0.20</td><td>25.98 ± 13.73</td><td>0.000</td></tr>
<tr><td>PT INR</td><td>1.03 ± 0.07</td><td>2.85 ± 1.17</td><td>0.000#</td></tr>
</table>

Data represent either n per group or mean ± SD, and differences between groups were tested using Student's t-test or Mann-Whitney U test #. Abbreviations: MELD score, model for end-stage liver disease score; CTP score, Child-Turcotte-Pugh score; AST, aspartate aminotransferase; ALT, alanine aminotransferase; PT INR, prothrombin time international normalized ratio.

Supplementary Table 2. The sequences of primers

Genes symbols (Human)	Forward	Reverse
GNA12	CTCAAGGGCTCAAGGGTTCTT	CAGGAACATCCCATGCTTCTC
ALOX12	GATCCCCCATATCCGCTACAC	CCACCTGTGCTCACTGCCTTAT
GAPDH	GAAGGTGAAGGTCGGAGTC	GAAGATGGTGATGGGATTTC
**Genes symbols (Mouse)**	**Forward**	**Reverse**
Gna12	TGCTTCGACGGCATCACA	GAAGTGCTTCTTAATGCTCACA
Hspa5	TGGTATTCTCCGAGTGACAGC	AGTCTTCAATGTCCGCATCC
Xbp1s	GAGTCCGCAGCAGGTG	GTGTCAGAGTCCATGGGA
Il1b	GGAGAACCAAGCAACGACAAAATA	TGGGGAACTCTGCAGACTCAAAC
Tnfa	TACTGAACTTCGGGGTGATCGGTCC	CAGCCTTGTCCCTTGAAGAGAACC
Il6	TTCCATCCAGTTGCCTTCTT	ATTTCCACGATTTCCCAGAG
Arg1	CTCCAAGCCAAAGTCCTTAGAG	AGGAGCTGTCATTAGGGACATC
Mrc1	CTCTGTTCAGCTATTGGACGC	CGGAATTTCTGGGATTCAGCTTC
Chil3	CAGGTCTGGCAATTCTTCTGAA	GTCTTGCTCATGTGTGTAAGTGA
β-actin	CTGAGAGGGAAATCGTGCGT	TGTTGGCATAGAGGTCTTTACGG
18S rRNA	GTAACCCGTTGAACCCCATT	CCATCCAATCGGTAGTAGCG
**microRNAs**	**Forward**	**Reverse**
miR-15a	TAGCAGCACATAATGGTTTGTG	Universal reverse primer (Qiagen proprietary information)
U6	GGGCAGGAAGAGGGCCTAT	
